# Interspecific and intraspecific foraging differentiation of neighbouring tropical seabirds

**DOI:** 10.1186/s40462-021-00251-z

**Published:** 2021-05-26

**Authors:** R. E. Austin, F. De Pascalis, S. C. Votier, J. Haakonsson, J. P. Y. Arnould, G. Ebanks-Petrie, J. Newton, J. Harvey, J. A. Green

**Affiliations:** 1 School of Environmental Sciences, University of Liverpool, Liverpool, L69 3GP UK; 2 Present Address: Department of Environmental Science and Policy, University of Milan, Milan, Italy; 3 The Lyell Centre, Heriot-Watt University, Edinburgh, EH14 4AP UK; 4 Department of Environment, Cayman Islands Government, George Town, Grand Cayman, KY1-1002 Cayman Islands; 5 School of Life and Environmental Sciences, Deakin University, Burwood, VIC 3125 Australia; 6 NERC National Environmental Isotope Facility, Scottish Universities Environmental Research Centre, Scottish Enterprise Technology Park, East Kilbride, G75 0QF UK; 7 Present Address: Guy Harvey Ocean Foundation, George Town, Grand Cayman, KY1-1005, Cayman Islands

**Keywords:** Red-footed booby, Brown booby, Competition, Resource partitioning, Foraging ecology

## Abstract

**Background:**

Social interactions, reproductive demands and intrinsic constraints all influence foraging decisions in animals. Understanding the relative importance of these factors in shaping the way that coexisting species within communities use and partition resources is central to knowledge of ecological and evolutionary processes. However, in marine environments, our understanding of the mechanisms that lead to and allow coexistence is limited, particularly in the tropics.

**Methods:**

Using simultaneous data from a suite of animal-borne data loggers (GPS, depth recorders, immersion and video), dietary samples and stable isotopes, we investigated interspecific and intraspecific differences in foraging of two closely-related seabird species (the red-footed booby and brown booby) from neighbouring colonies on the Cayman Islands in the Caribbean.

**Results:**

The two species employed notably different foraging strategies, with marked spatial segregation, but limited evidence of interspecific dietary partitioning. The larger-bodied brown booby foraged within neritic waters, with the smaller-bodied red-footed booby travelling further offshore. Almost no sex differences were detected in foraging behaviour of red-footed boobies, while male and female brown boobies differed in their habitat use, foraging characteristics and dietary contributions. We suggest that these behavioural differences may relate to size dimorphism and competition: In the small brown booby population (*n* < 200 individuals), larger females showed a higher propensity to remain in coastal waters where they experienced kleptoparasitic attacks from magnificent frigatebirds, while smaller males that were never kleptoparasitised travelled further offshore, presumably into habitats with lower kleptoparasitic pressure. In weakly dimorphic red-footed boobies, these differences are less pronounced. Instead, density-dependent pressures on their large population (*n* > 2000 individuals) and avoidance of kleptoparasitism may be more prevalent in driving movements for both sexes.

**Conclusions:**

Our results reveal how, in an environment where opportunities for prey diversification are limited, neighbouring seabird species segregate at-sea, while exhibiting differing degrees of sexual differentiation. While the mechanisms underlying observed patterns remain unclear, our data are consistent with the idea that multiple factors involving both conspecifics and heterospecifics, as well as reproductive pressures, may combine to influence foraging differences in these neighbouring tropical species.

**Supplementary Information:**

The online version contains supplementary material available at 10.1186/s40462-021-00251-z.

## Background

Understanding how coexisting species and individuals use and partition resources is central to knowledge of community structure in wild populations [1, 2], and a key component for identifying conservation priorities [3–5]. Consumers must adopt highly efficient strategies to acquire ample resources for survival and reproduction [6, 7]. Thus, it is often advantageous for animals to develop behaviours that minimise conflict with others [2]. For example, where multiple species with similar morphologies coexist, this can manifest as resource partitioning in space, time and/or diet, resulting in divergent ecological niches [1, 8–11].

Such pressures and outcomes also operate within species, and intraspecific segregation in resource use based on sex, life stage and even at the individual level is common within the animal kingdom [12–14]. Such resource partitioning has been widely associated with factors linked to body size differences [15, 16], and in communities with large populations may be driven by density dependence [17, 18]. While competitive pressures offer one potential explanation for interspecific and intraspecific differentiation in foraging, many other factors such as differing nutritional or physiological requirements [19], predation risk [4, 20], or sociality (e.g. avoidance of mating attempts: [21, 22]) have been proposed as causal factors, although a limited consensus exists between studies and systems.

For highly mobile marine vertebrates constrained to breed on land, such as seabirds, operating successfully within ocean systems is fraught with challenges. Access to suitable nesting habitat and widely-distributed prey can limit population processes [23–25], and these influences can become particularly pronounced during breeding periods when movements of central-place foragers are constrained in space and time [26]. Throughout the global oceans, these challenges result in the coexistence of multiple colonial seabird species within ecosystems, and thus in varied forms of ecological segregation [10, 11, 19]. Nevertheless, some communities in highly productive systems that offer abundant resources appear to lack niche divergence between their constituent species (i.e. [27, 28]).

In tropical and subtropical oceans, our understanding of factors that affect foraging differentiation and community structure lags behind that for many other regions [29, 30]. Yet these environments, characterised by low productivity and limited seasonal variability [31], support diverse communities of marine vertebrates including large populations of seabirds [32]. In comparison to the impressive dive depths common amongst temperate and polar seabirds, many tropical species feed at or near the ocean’s surface, where social and commensal foraging in mixed aggregations is common [33–35]. This propensity for co-exploitation of resources contrasts with predictions of ecological niche divergence, and highlights a need for improved knowledge of multi-species interactions in these systems.

Two congeneric tropical seabirds, the red-footed booby (*Sula sula*, Linnaeus, 1766; hereafter referred to as the RFB) and brown booby (*S. leucogaster*, Boddaert, 1783; hereafter referred to as BB), commonly co-exist on islands throughout the tropics [36–38]. These species share similar morphological traits, the RFB being slightly smaller and more slender than the BB, yet exhibit striking differences in breeding behaviour [36, 37]: RFBs are arboreal nesters while BBs predominantly employ a ground-nesting strategy [37]. To be successful, these species must deal not only with constraints associated with securing suitable nest sites, but those imposed within the foraging environment in which they operate [39]. Thus, understanding the mechanisms by which these sulids coexist requires consideration of factors in both marine and terrestrial habitats. While RFBs and BBs have received considerable attention for tropical species, with some interspecific differences in foraging ecology reported [35, 40–44], the degree to which they coexploit and/or partition marine resources, both in terms of space use and diet, remains poorly understood [36, 45].

Here, we investigated whether coexisting populations of these two tropical species have evolved divergent foraging behaviour with high levels of segregation at sea, mirroring their separation in nesting habitat, or whether they overlap in their resource use. To answer this question, we studied interspecific and intraspecific differences in the spatial movements, dive behaviour, activity patterns, social interactions and diet of two neighbouring populations that breed contemporaneously in the Caribbean Sea. The Cayman Islands archipelago in the Western Antilles has resident populations of both species that nest in close proximity on neighbouring islands, yet differ in their population sizes. The RFB booby population is over an order of magnitude larger than the BB population, and co-occurs at its nesting site with breeding magnificent frigatebirds, thus experiencing regular kleptoparasitic pressure from this predator. We hypothesise that the close proximity of the BB and RFB populations, and differences in both their densities and risk of kleptoparasitism, will introduce pressures that manifest through divergent foraging behaviours and/or dietary preferences within their environment.

## Methods

### Study site and bio-logging

**Fig. 1:**
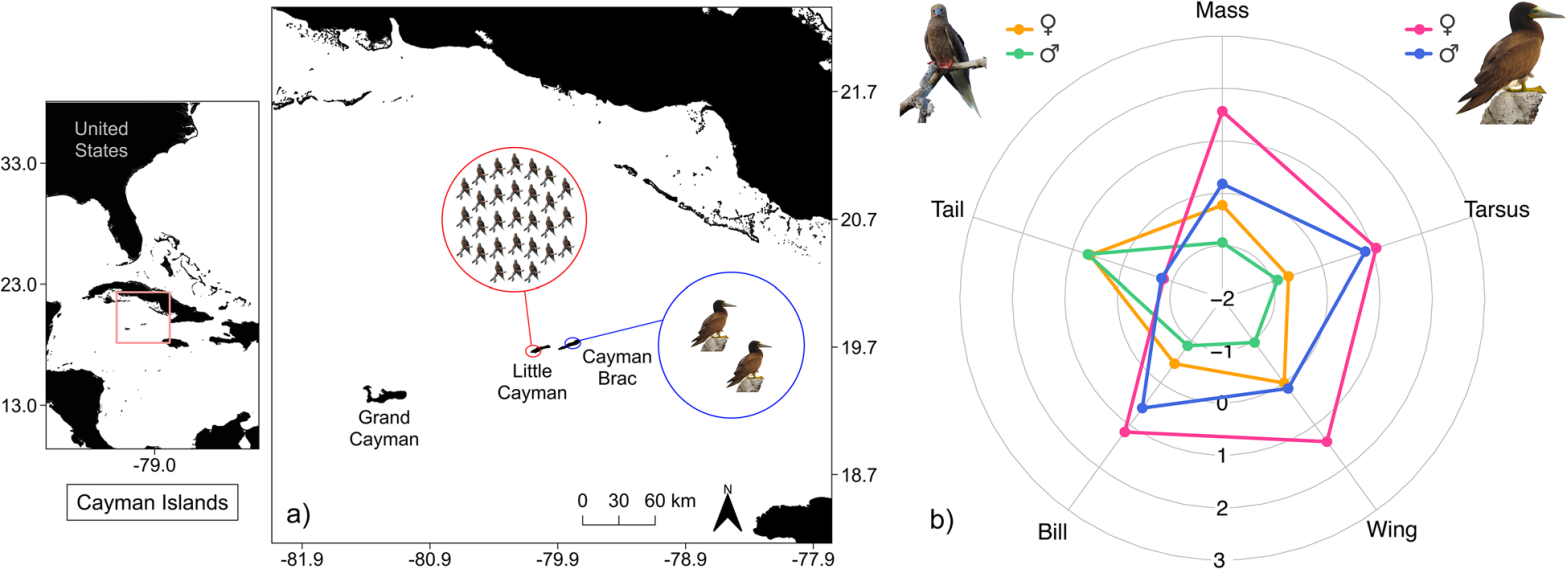
**a** Colony locations of red-footed boobies (population size >2000 indiviudals) and brown boobies (population size <200 individuals) on the Cayman Islands and **b** A radial plot showing standardised mean body size and mass measurements (*x* - mean / sd, range: -1 to +1) for both species by sex (*n*, red-footed boobies: female/orange = 28, male/green = 41; brown boobies: female/pink = 25, male/blue = 33). Effect sizes and results of statistical comparisons of morphometrics within and between species are presented in Additional file 1: Table S3

Data were collected from two closely-situated populations of boobies that breed at neighbouring sites (islands ~ 7 km apart, nests ~ 26–39 km apart) on the Cayman Islands in the Caribbean Sea: 1) the Booby Pond Nature Reserve on Little Cayman, a RAMSAR site that hosts an internationally important breeding population of RFBs (Fig. 1; Latitude: 19.663 °N, Longitude: 80.082 °W; estimated population size in 2017: 2094 breeding adults, [46]); and 2) beach and cliff locations on Cayman Brac that support a small scattered breeding population of BBs (Fig. 1; Latitude: 19.711 °N, Longitude: 79.801 °W; estimated population size in 2017: 146 breeding adults, [47]).

All fieldwork was performed under permissions of the Department of Environment, Cayman Islands Government and/or National Trust of the Cayman Islands, following established protocols to minimize disturbance. All handling procedures were undertaken following ethical guidelines of the Universities of Liverpool and Exeter. To assess the impact of device attachment on the reproductive performance of tagged animals, the fledgling success (measured as the proportion of nests that hatched and fledged a chick) was recorded in experimental nests and unhandled closely matched control nests dispersed throughout the colonies. Fisher’s exact tests were used to test for significant differences between groups.

During chick-rearing (Feb - April) between 2016 and 2019, RFBs (*n* = 31) and BBs (*n* = 68) were tracked with archival GPS loggers (Mobile Action iGotU GT-120 s; Mass = ~ 15 g; chick age range = 2 – 13 weeks, see Additional file 1, Appendix S1 for further details), set to record at intervals of either 30 s or 2 min. Incorporation of interpolated tracks originally recorded at ~ 2-min intervals had no notable effect on spatial analyses (Additional file 1, Appendix S2). Devices were attached to a small number of back contour feathers using waterproof tape, and were recovered after at least one foraging trip. A subset of boobies were simultaneously tracked in 2017, 2018 and 2019 with time-depth recorders to record dive activity (TDRs - Cefas Technology G5s; Mass = 2.5 g; Sampling interval = 1 Hz; *n*, RFBs = 20, BBs = 27), and immersion loggers to measure on-water activity (Migrate Technology C65s; Mass = 1 g; *n*, RFBs = 17, BBs = 15). Immersion loggers, set to record changes from wet to dry states every 6 s, were attached to a plastic ring on the tarsus, while TDRs were attached to the underside of the two central tail feathers using waterproof tape.

To assess the presence, rate and behavioural context of kleptoparasitic interactions with magnificent frigatebirds, 16 brown boobies (9 females, 7 males) were instrumented with a miniaturised video data logger in 2018 (Catnip Technologies, Hong Kong; Mass = 24.7 – 26.7 g). Twelve of these individuals (8 females, 4 males) were also tracked simultaneously with a GPS logger to obtain matching spatial locations (see above). Loggers were set to record for 30 min periods every 2 h during daylight (cumulative recording time of ~ 4 h). The total mass of combined loggers in the study did not exceed 3% body mass (Mean % body mass, BBs = 2.4 ± 0.8 g, RFBs = 2.3 ± 0.4 g), with the exception of 14 BBs that were fitted with either a video logger or accelerometer for a simultaneous study (in these cases device mass never exceeded 4.5% body mass). RFBs were not tracked with video loggers owing to size constraints.

Birds were weighed prior to device deployment, and a range of morphometric measurements, including flattened wing length, bill length, bill depth, bill width, tarsus length and tail length, were taken with dial calipers (± 0.01 mm) or a steel rule (± 0.1 mm) by the same researcher to determine body size. As the sex of RFBs cannot be reliably determined in the field, DNA sexing was undertaken on a subset of sampled birds (*n* = 69) using blood samples or three to four breast feathers collected during handling (Animal Genomics Laboratories, UK). The sex of birds that tissue was not extracted from (*n* = 10) was predicted based on results of a discriminant function analysis undertaken on morphometric data from birds of known sex (see Additional file 1, Appendix S3).

### Dietary habits

To investigate trophic habits, carbon and nitrogen stable isotope values in blood samples of foraging birds were analysed (*n*, RFBs 2016 = 37, 2017 = 22; BBs 2016 = 11, 2017 =19). Blood was sampled from the tarsal vein of tracked individuals upon first capture, using a needle and syringe, and spun in a centrifuge for 15 min to extract red blood cells (RBCs) for analysis, before being frozen. RBCs were dried in an oven at low temperatures (35 – 40 °C) until reaching constant mass, ground into a powder and weighed into tin capsules in preparation for stable isotope analysis (0.5 – 0.8 mg).

A range of fish and squid prey species were sampled opportunistically from regurgitates of tracked birds (RFBs = 15, BBs = 30). To examine diet, samples were identified to the lowest taxonomic level possible, and subsequently analysed to determine stable isotope compositions. Small sections of dorsal white muscle tissue (~ 2 cm) were extracted, dried, ground and weighed into capsules following methods outlined above. To account for contributions from ^13^C-depleted lipids in fish muscle samples, lipid extracted δ^13^C values were predicted using lipid-normalisation equations from [48] (following methods in [49]).

Stable isotope analysis was performed at the Natural Environment Research Council Life Science Mass Spectrometry Facility, East Kilbride in 2016, and the University of Liverpool School of Environmental Sciences Isotope laboratory in 2017, using continuous-flow isotope mass spectrometry. Isotope ratios were expressed in δ notation in parts per thousand (‰) relative to V-PDB (δ^13^C) or AIR (δ^15^N) scales. Multiple measurements of internal laboratory standards indicated that measurement error was ≤0.1 ‰ for both δ^13^C and δ^15^N.

### Data analysis

Less than 0.01% of GPS locations for BBs and 0.03% for RFBs were associated with ground speeds of > 95 km h^− 1^ (consistent with existing reports of instantaneous flight speeds in these or similar species: [50–52]). Therefore, we filtered GPS locations for unrealistic speeds above this threshold. Prior to further processing, raw GPS data were also filtered to remove partial trips, colony-based movements (< 500 m from nest) and movements away from the colony < 30 min in duration.

To allow a direct comparison of foraging distributions between species, Hidden Markov Models (HMMs), based on step lengths and turn angles, were trained to estimate behavioural states in tracks using the ‘momentuHMM’ package in R [53]. Prior to fitting models, GPS locations were interpolated to 30 s intervals using cubic piecewise hermite polynomials (following [54]), and colony-based locations were removed. Step lengths were modelled using a gamma distribution, while turn angles were modelled with a von Mises distribution. HMMs were validated using dive and immersion data from birds tracked with simultaneously deployed TDRs and immersion loggers (see Additional file 1, Appendix S4 for details). Appropriate parameter priors for the final model were selected through a comparison of negative log-likelihood values of a number of candidate models run iteratively using a range of randomly selected mean and SD parameter values constrained within realistic limits (*n* = 25). Following the assignment of time points to behavioural states, all locations estimated to be associated with directed flight and rest were discarded, and bouts of movement associated with foraging were extracted to map density distributions.

Fixed Kernel Density Estimates (KDEs) were calculated on HMM-estimated foraging data. To prevent spatial biases, covariance bandwidth matrices were obtained using the least squares cross validation estimator (‘ks’ package in R, [55]) on projected coordinates. The overlap between kernel density estimates (50 and 90% KD contours representing the core and main foraging areas) of different sexes and species was calculated using Bhattacharyya’s affinity [56]. Intra-annual comparisons of the core (50% KDE) and main (90% KDE) foraging areas for 2016 and 2017 (when both species were tracked) indicated that differences in space use between species were consistent across sample years (Fig. S3; Bhattacharyya’s affinity, 2016: 50% = 0, 90% = < 0.1; 2017: 50% = 0, 90% = < 0.01). Thus, we pooled all data across years for comparison of species distributions. For each foraging track, total distance travelled, maximum distance from colony, trip duration, mean distance from the nearest coastline, median underlying bathymetry (obtained via the marmap package in R: [57]) and time spent in different behavioural states (see below) were calculated. Mixed-effects models with a random individual intercept were run to compare trip characteristics between species and sexes.

To investigate the presence of different foraging tactics, we firstly used a PCA to extract appropriate variables for further behavioural clustering (see Additional file 1, Appendix S5). To identify clustering in the data, Gaussian Mixture Models were run on trips from both species using ‘trip duration’, ‘distance to nearest coastline’ and ‘maximum distance’ parameters. As BB trips clustered into two groups, we used Binomial GLMMs with a random intercept for individual on this species, to investigate differences in the probability of foraging coastally versus pelagically between the sexes.

Dives were classified using the ‘diveMove’ package in R [58]. Depth measurements were calibrated using a ‘moving quantile’ zero-offset correction method (following [59]) and a dive threshold of > 0.25 m. Dive and immersion data were matched to the nearest spatial location obtained from 30 s interpolated GPS data, and dive metrics were calculated within 30 s segments of track centred on each location for all subsequent spatial analyses. The mean dive rate (no. dives hr^− 1^) of each species was calculated and mapped within 5 km × 5 km grid cells.

Video footage was analysed frame-by-frame (~ 30 frames・s^− 1^) using VirtualDub software (Avery Lee), and behaviour of the tagged bird was categorised for each second using a specifically designed ethogram. All data were analysed by a single observer and validated by an independent observer. For all kleptoparasitic interactions, we recorded time, duration, and the sex and age class of the attacking frigatebird. Interactions were considered discrete if there was a gap of 30 s. We also recorded the time of interactions with respect to the time when boobies were searching/foraging or engaging in prey capture. We compared differences in the proportion of male and female boobies targeted with a Fisher’s exact test, and plotted the spatial distribution of kleptoparasitic interactions within 30 s curvilinear interpolated GPS data from tracked birds. Distance to nearest coastline, and the number of kleptoparasitic events within 5 km × 5 km grid cells over the foraging range of video-instrumented birds, were determined. Departure and arrival times to and from the colony (< 500 m from nest sites) were calculated from GPS data.

The isotope niche spaces occupied by sampled birds and their prey were estimated using standard ellipse areas (corrected for small sample sizes: SEAc) calculated in the SIAR package in R [60]. As isotopic discrimination factors between blood and prey muscle tissue have not been published for Sulids, the mean and standard deviations of discrimination factors for similar species in the literature (Additional file 1, Table S7) was applied to avian data to allow a comparison with reference prey data. Differences in bulk carbon and nitrogen isotope values between sexes, species and years were tested with generalised least squares models (weighted linear regression; GLS), with an added variance structure to allow for different variances per factor level. Repeated isotope values between years were sampled from only one BB, and only the first measure was used for this individual during modelling. Morphometric measurements of species and sexes were compared using either linear models or GLS models with variance structures for species or sex in cases of unequal variances between factor levels.

## Results

### Device effects

There was no significant difference between the fledgling success of experimental nests and control nests for the two study species, with the exception of RFBs in 2017 when control pairs had lower fledging success than experimental pairs (Additional file 1, Table S8). This suggests that handling and tagging disturbance had no notable detrimental effect on the ability of experimental birds to successfully raise a chick.

### Body mass and size

Body mass and size differed significantly between species, and between sexes within species. BBs were heavier and larger than RFBs in all measures (Mass, Bill length, Tarsus length: GLS, *p* < 0.001; Wing length: LM, *p* < 0.001), except tail length which was longer in the latter species (GLS, *p* < 0.001, Fig. 1 & Table S3). Females of both sexes were also heavier and larger than males (GLS, *p* < 0.001), with the exception of tail length which did not differ with sex (GLS, *p* = 0.681, Fig. 1 & Table S3), and the degree of size dimorphism was greater in BBs than RFBs for most metrics (Cohen’s *d* effect sizes all > 0.6, except for tail length comparisons where *d* < 0, and the BB tarsus length comparison where *d* = 0.4; see Table S3).

### Interspecific and intraspecific partitioning of movement

**Fig. 2:**
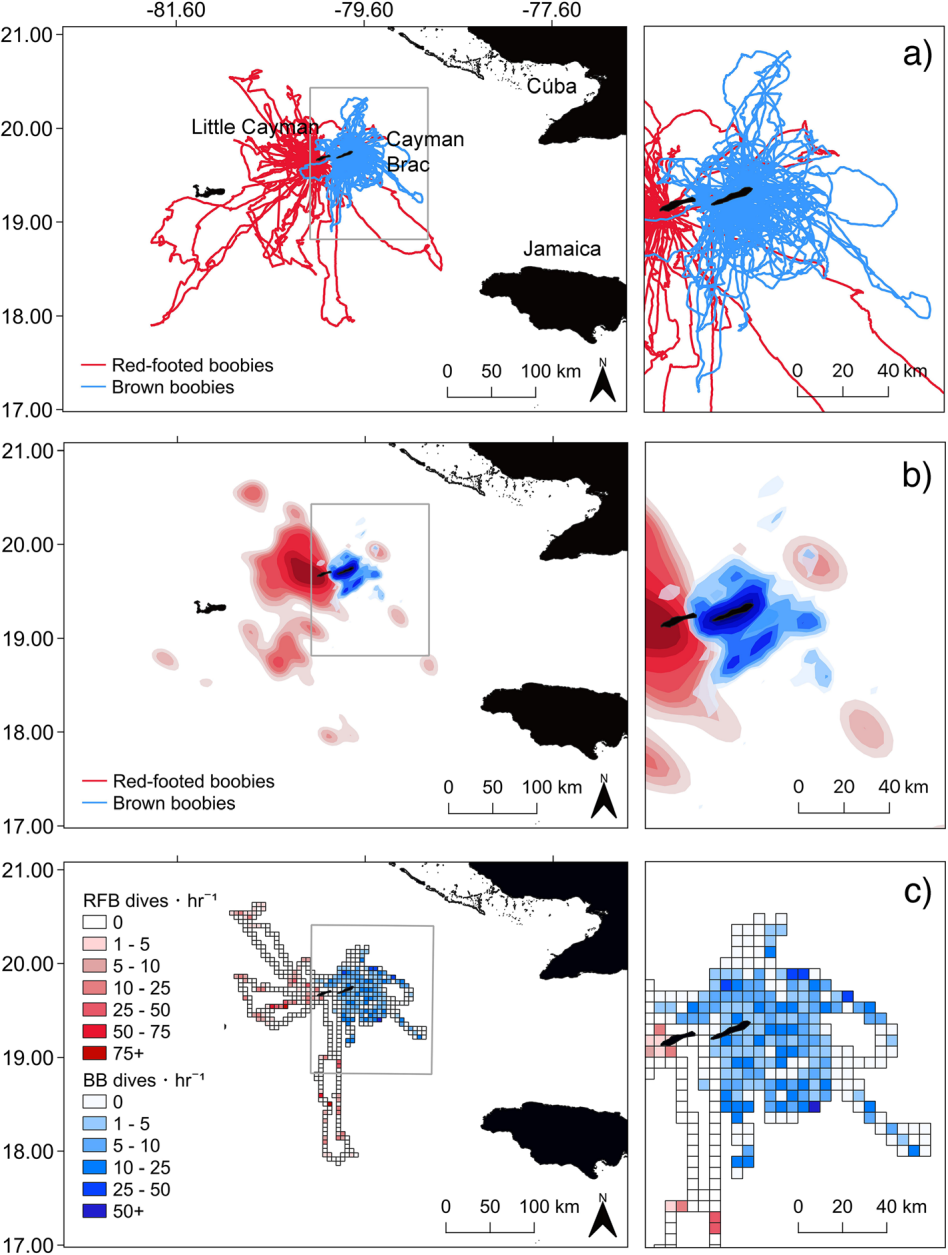
Foraging and dive distributions of red-footed boobies and brown boobies tracked from the Cayman Islands **a** Foraging tracks, **b** Kernel density distributions of foraging locations classified with Hidden Markov Model, and **c** Dive distributions (mean bird dives hr^− 1^) of red-footed boobies (*n*, GPS = 24, TDR = 8) and brown boobies (*n*, GPS = 58, TDR = 18), tracked with biologgers from neighbouring populations in the Cayman Islands during breeding seasons between 2016 and 2019

Between 2016 and 2019, 217 full foraging trips from 58 BBs (13 partial) and 54 full trips from 24 RFBs (14 partial; Fig. S6) were recorded. For these GPS-tracked birds, 18 dive and 13 immersion traces were obtained for BBs, while eight dive and 10 immersion traces were obtained for RFBs (see Table S9 for a full summary of deployments and recoveries). On average, RFBs travelled significantly further from the nest, foraged in deeper waters, had larger home ranges, and spent longer periods at sea than the more coastal short-ranging BBs (Fig. 2, Table 1). Both species exhibited shallow dive behaviour, with foraging occurring almost exclusively within the top 2 m of ocean (BBs = 98%, RFB = 99%; Table 1). BBs dived on average to greater depths than RFBs, however, differences in depth were small (< 30 cm on average), and no differences in dive rate or duration were detected (Table 1; Fig. S7). While RFBs often spent the entire day at sea (or multiple days, rafting at night; 60% trips > 8 h in duration), commonly departing and returning to the colony during crepuscular hours, BBs were exclusively diurnal foragers with more variable departure and return timings (Fig. S8). The two species showed high levels of spatial segregation with almost no overlap in both their core and main foraging areas (Overlap: 50% occupancy kernels = 0, 90% occupancy kernels = 0.02). No differences were found in time spent engaging in different behaviours between species nor sexes, with the exception of higher travel time in female RFBs (Table 1).

**Table 1:** Foraging trip characteristics (mean ± SE) of red-footed and brown boobies by sex, and results of GLMMs

	Red-footed boobies			Brown boobies			*χ*^2^_1_ (*p*)		
Parameter	Female	Male	All	Female	Male	All	Sex RFB	Sex BB	Species
*n* (birds / trips)	5 / 8	19 / 46	24 / 54	27 / 124	31 / 93	58 / 217	–	–	–
Max dist. (km)	90.4 ± 34.0	57.4 ± 6.9	62.3 ± 7.7^b^	14.8 ± 1.4^a^	23.5 ± 1.7^a^	18.5 ± 1.1^b^	1.191 (0.275)^g^	**7.496 (0.006)**	**22.491 (< 0.001)**
Dist. coast (km)	44.0 ± 17.0	31.8 ± 0.3	33.6 ± 3.8^b^	4.5 ± 0.9^a^	11.3 ± 0.1^a^	7.4 ± 0.7^b^	0.539 (0.463)	**6.298 (0.012)**	**28.91 (< 0.001)**
Total dist. (km)	252.1 ± 83.4	194.5 ± 22.9	203.0 ± 22.8^b^	49.9 ± 3.9^a^	77.2 ± 5.5^a^	61.6 ± 3.4^b^	0.472 (0.492)	**5.729 (0.017)**	**26.33(< 0.001)**
Trip dur. (h)	14.6 ± 5.4	12.4 ± 1.8	12.7 ± 1.7^b^	3.0 ± 0.3^a^	4.4 ± 0.4^a^	3.6 ± 0.3^b^	0.041 (0.840)^g^	**4.311 (0.038)**^**g**^	**21.40 (< 0.001)**
Prop. time forage*	0.41 ± 0.03	0.52 ± 0.02	0.50 ± 0.02	0.50 ± 0.02	0.50 ± 0.02	0.50 ± 0.01	3.807 (0.051)	0.141 (0.708)	0.230 (0.631)
Prop. time travel*	0.39 ± 0.03	0.29 ± 0.01	0.31 ± 0.01	0.32 ± 0.02	0.36 ± 0.02	0.34 ± 0.01	**4.813 (0.028)**	0.005 (0.945)	1.448 (0.229)
Prop. time rest*	0.23 ± 0.04	0.19 ± 0.02	0.20 ± 0.02	0.21 ± 0.02	0.17 ± 0.02	0.20 ± 0.01	0.885 (0.347)	1.653 (0.199)	0.964 (0.326)
Core HR (km^2^)	143.5 ± 66.3	61.2 ± 11.3	73.4 ± 14.0^b^	15.3 ± 3.4	30.4 ± 4.1	21.8 ± 2.7^b^	1.550 (0.213)	1.034 (0.309)	**9.197 (0.002)**
Main HR (km^2^)	827.3 ± 424.9	335.7 ± 71.9	408.5 ± 88.6^b^	58.2 ± 11.7	121.2 ± 14.4	85.2 ± 9.3^b^	1.366 (0.242)	1.853 (0.173)	**0.704 (0.001)**
Bathymetry (m)	2023 ± 476.0	2321 ± 209	2277 ± 190^b^	793 ± 98^a^	1634. ± 125^a^	1153 ± 82^b^	0.362 (0.548)	**12.342 (< 0.001)**	**13.043 (< 0.001)**
Dives hr.^−1^	8.8 ± 2.8	6.7 ± 1.4	7.4 ± 1.3	7.2 ± 1.1	7.2 ± 0.9	7.2 ± 0.7	< 0.001 (0.993)	0.027 (0.868)	0.991 (0.970)
Max dive depth (m)	0.42 ± 0.03	0.50 ± 0.05	0.47 ± 0.03^b^	0.73 ± 0.05	0.73 ± 0.04	0.73 ± 0.03^b^	0.388 (0.534)	0.013 (0.909)	**16.219 (< 0.001)**
Dive dur. (s)	5.8 ± 1.6	4.2 ± 1.7	4.8 ± 1.3	5.1 ± 2.2	2.1 ± 0.3	3.7 ± 1.2	0.591 (0.442)	1.702 (0.192)	1.171 (0.279)

Mean (±SE) foraging trip characteristics and parameters from generalised linear mixed-effects models (GLMMs), of chick-rearing red-footed boobies and brown boobies tracked from neighbouring populations in the Cayman Islands during breeding seasons between 2016 and 2019. Unless otherwise indicated, GLMMs were specified with a random ‘individual’ intercept and either a Gamma error distribution^(g)^, or Gaussian distribution with a variance structure to allow the variance to vary by sex

*Dist. Coast* mean distance to nearest coastline, *Max dist.* maximum distance from nest, *Total dist.* total distance travelled, *Trip dur.* trip duration, *Prop. time forage/rest/travel* proportion of trip time spent in behaviour, *HR* home range, *Dive dur.* dive duration

*Beta-binomial GLMMs with a logit link. Shared superscript letters within each parameter indicate significant differences between ^a^sexes and ^b^species (*p* < 0.05)

**Fig. 3:**
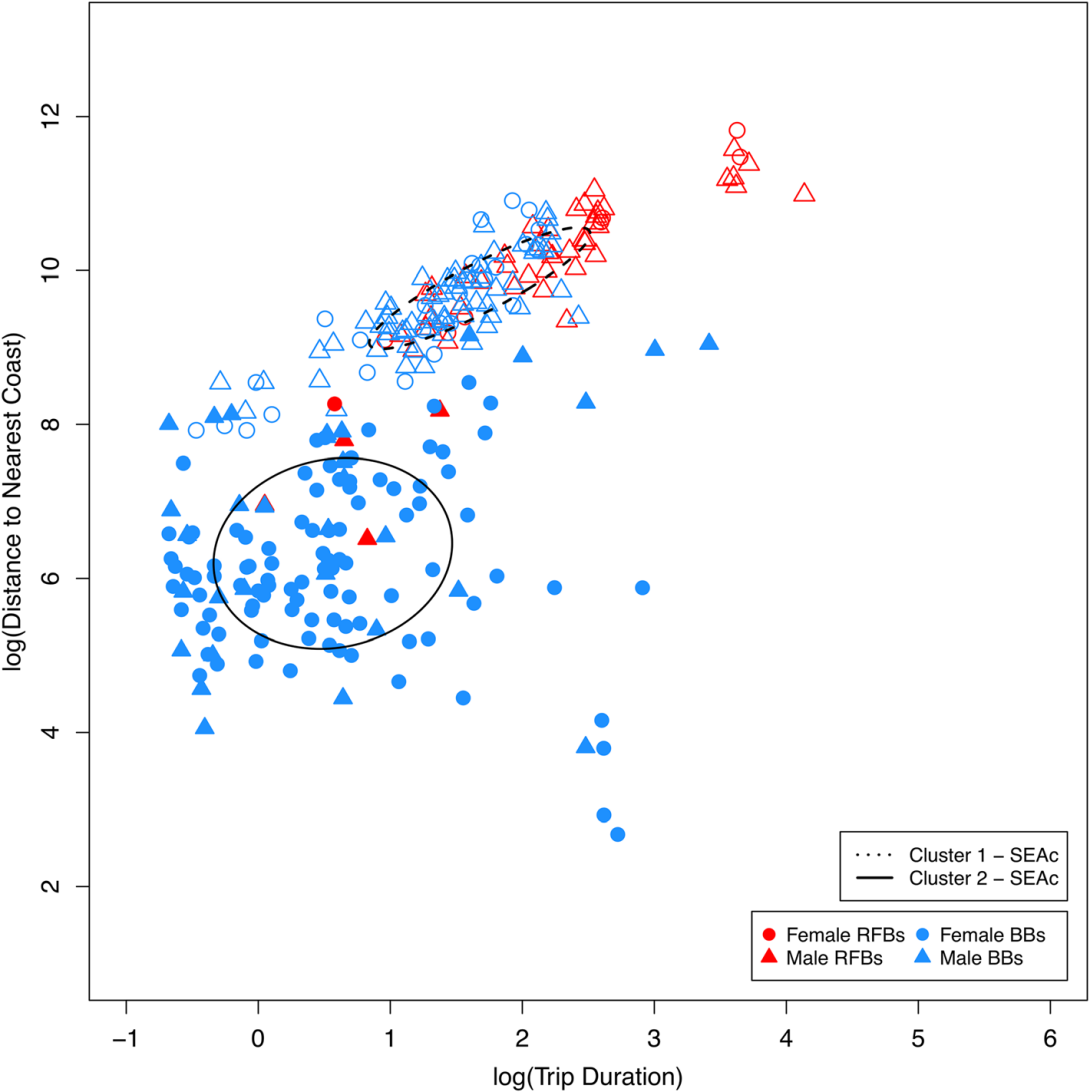
Foraging trips of red-footed boobies (red; *n* = 54) and brown boobies (blue; *n* = 217) displayed according to trip duration and distance from nearest coastline, and coloured according to one of two GMM-assigned clusters (open points: cluster characterised by longer trips further from coast, filled points: cluster characterised by coastal trips of shorter duration). Triangles = males, circles = females

Foraging trips fell into two main clusters (C1 and C2) based on ‘trip duration’, ‘distance to nearest coastline’ and ‘maximum distance’ (see Additional file 1), illustrating divergence in foraging tactics. Almost all RFB trips clustered together (C1,: 91%) and were characterised by longer trip durations further from shore (also correlated with greater underlying water depths, larger home ranges, and greater distances travelled). In contrast, BB trips were variable in their characteristics, falling into the two clusters: neritic shorter trips (C2) and more extensive pelagic trips of longer duration similar to RFBs (C1; Fig. 3). For BBs, males and females undertook both types of foraging trips, although males had a significantly higher probability of engaging in the longer, more extensive trip type than females (LRT, *χ*^2^_1_ = 21.299, *p* < 0.001; C1, 67% of male trips, 22% of female trips).

**Fig. 4:**
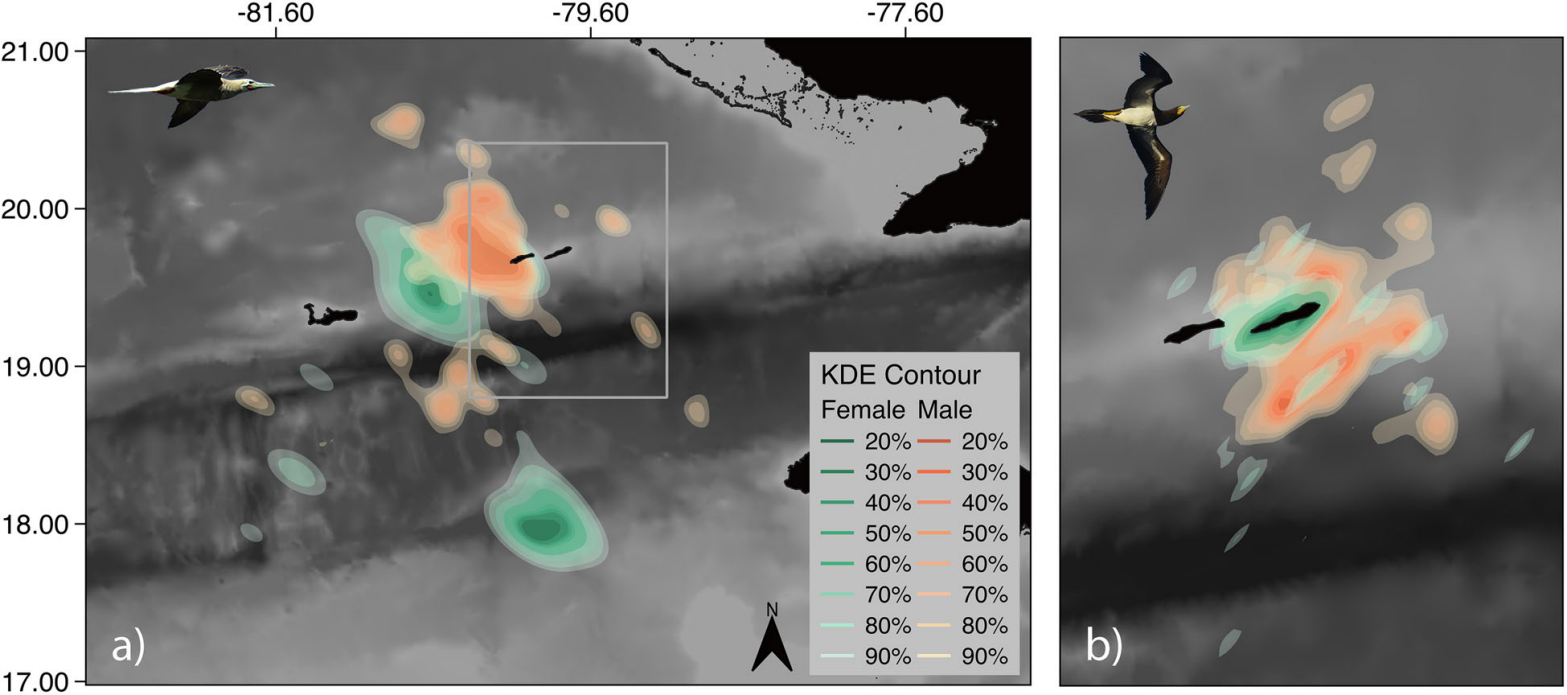
Foraging distributions of red-footed boobies and brown boobies according to sex. Kernel density distributions of foraging locations for male and female red-footed boobies (left panel; *n* bird/trips, female = 5/8, male = 19/46) and brown boobies (right panel; *n,* female = 27/124, male = 31/93) are shown. Males = orange, Females = green. Distributions are mapped over GEBCO 1 arc-second bathymetry data (source: GEBCO Digital Atlas, Intergovernmental Oceanographic Commission, International Hydrographic Organization and the British Oceanographic Data Centre)

Sex differences in spatial distributions and trip characteristics were marked in the highly size-dimorphic BB, the males of which undertook significantly longer trips than females, foraging further from the nest over deeper waters (Fig. 4 and Table 1). Intersexual differences in movements and trip characteristics were almost entirely absent in RFBs (Fig. 4 and Table 1).

### Kleptoparasitic interactions

**Fig. 5:**
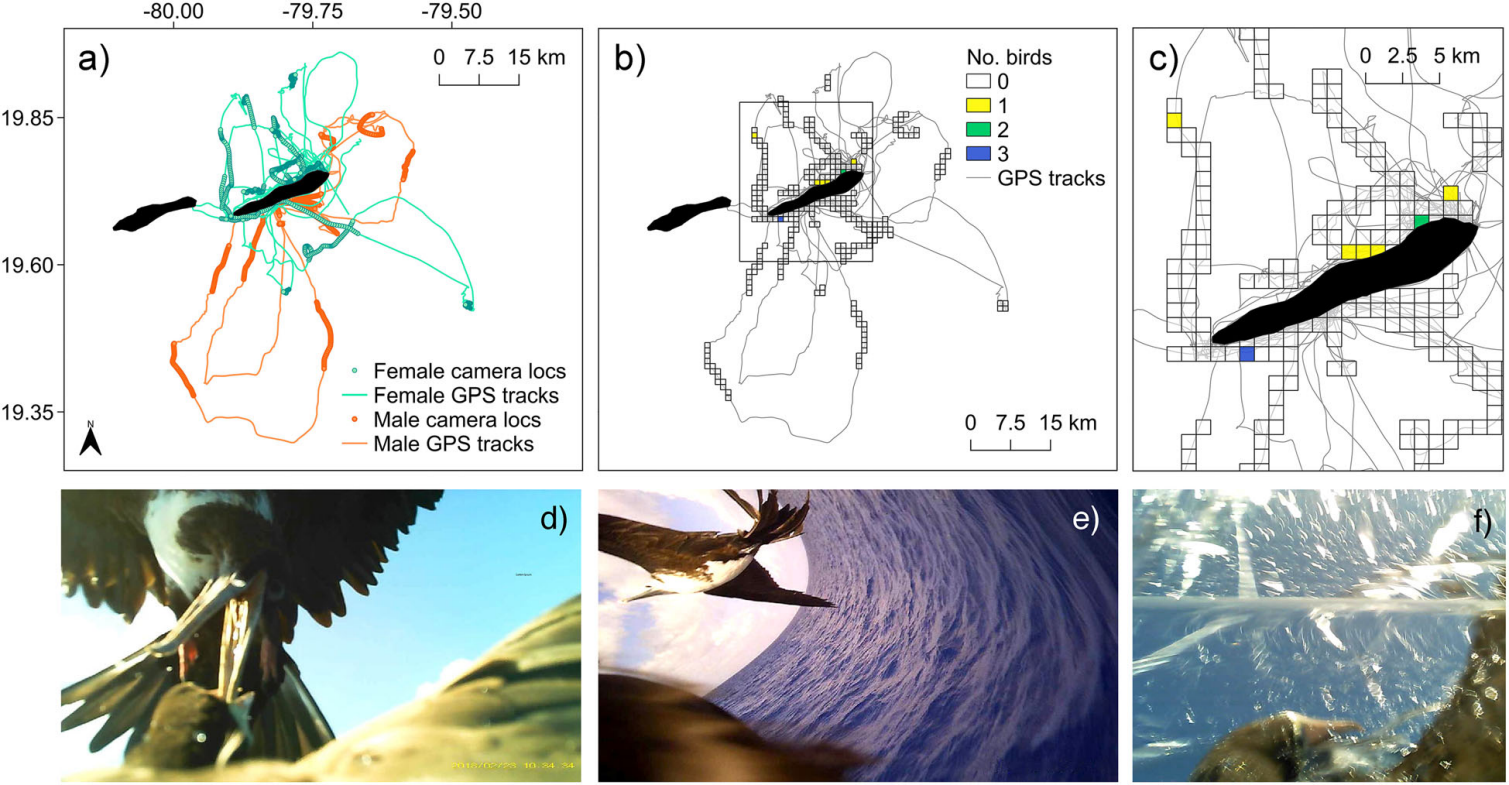
Distribution of kleptoparasitic interactions between magnificent frigatebirds and brown boobies **a** Foraging tracks of brown boobies that were simultaneously instrumented with video loggers and GPS from a population on the Cayman Islands during 2018. Tracks coloured according to sex (*n*, green/female = 7, orange/male = 4). Full tracks are shown with lines and sections of track containing matching video footage with circular points. **b** & **c** Number of individual boobies within 5 × 5 km grid cells that experienced kleptoparasitic interactions within video-tracked sections of foraging trips. **d** & **e** Example frames showing kleptoparasitic interactions and **f** A frame showing a booby pursuing prey underwater

Twelve kleptoparasitic interactions between frigatebirds and brown boobies (*n* = 5 individuals) were detected in 19.5 h of video data, totalling 3.78 min (interaction duration range = 4 – 45 s; Additional file 1, Appendix S9). Frigatebirds only kleptoparasitised female boobies (*n* = 5 of 9 females vs 0 of 7 males; Fisher’s exact test, *P* = 0.034), and all attacks were undertaken by adult female (*n* interactions = 10; Fig. 5) or juvenile frigatebirds (*n* interactions = 2; Fig. 5). There were no differences in mass between parasitized and non-parasitized females (targeted = 1313 ± 117 g, not targeted: 1323 ± 120 g; Wilcoxon rank-sum test, *W* = 8, *p* = 1).

All kleptoparasitic interactions observed on birds with matching spatial data (*n* interactions = 10; *n* birds = 11) occurred when the tracked booby was in coastal waters, with only one interaction occurring > 1.5 km from shore (Fig. 5). All kleptoparasitic interactions took place during booby searching and foraging activity, or soon before/after these behaviours (< 2.4 min; see Additional file 1, Fig. S9), although the success of the frigatebird was unclear. In two cases, the targeted booby was observed catching prey < 30 s from the start of the interaction (Fig. 5). See Additional file 2, Video S1 for example footage of a kleptoparasitic interaction.


**Additional file 2: Video S1.** Description of data: example video sequence showing kleptoparasitic interaction between a magnificent frigatebird and brown booby.

### Dietary partitioning

**Fig. 6:**
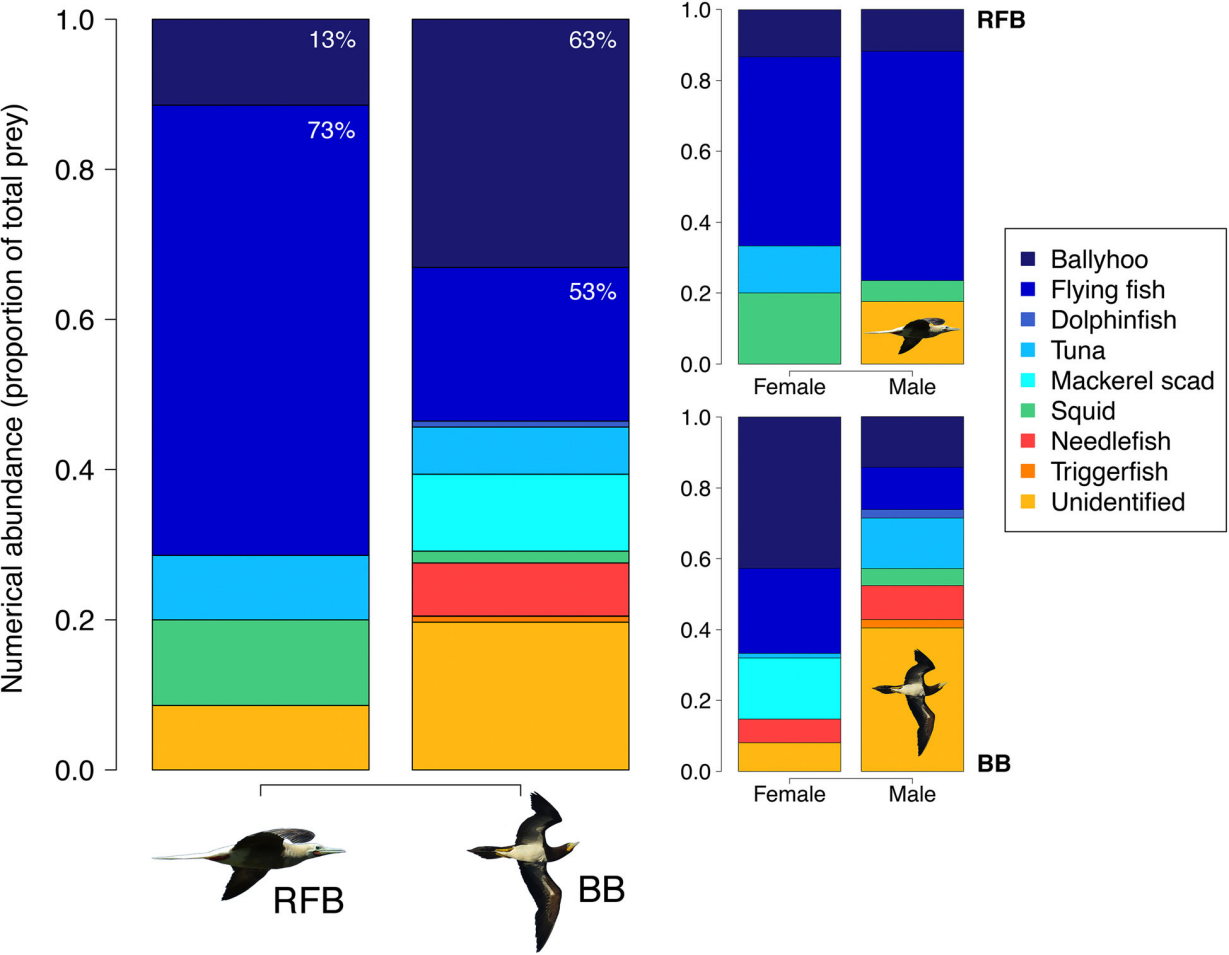
The numerical abundance of different prey types in regurgitate samples (expressed as the proportion of each prey type out of total prey sampled) of tracked red-footed boobies (RFB *n* = 15) and brown boobies (BB *n* = 30) from colonies on the Cayman Islands, during chick-rearing periods between 2016 and 2018. For each species, data are shown for all individuals combined (left sub-figure), and according to sex (right sub-figures; RFB *n*, female = 6, male = 6; BB *n*, female = 18, male = 9). Values on bars show the frequency of occurrence (percentage of birds with a prey type present in their regurgitate) of flying fish and ballyhoo

The 45 regurgitates collected (*n* birds, BBs = 30, RFBs = 15) contained 196 individual prey samples identifiable to at least the family level. Ballyhoo (family: Hemiramphidae) and flying fish (family: Exocoetidae) were most abundant overall. RFBs ate more flying fish, while BBs ate more ballyhoo, additionally consuming a small number of inshore and reef-associating species including triggerfish (family: Balistidae) and needlefish (family: Belonidae) (Chi-squared test, *χ*^2^ = 21.363, *df* = 2, *p* < 0.001; Fig. 6 & Table S11). For RFBs, 27% regurgitates contained ≥2 prey types, while for BBs 46% regurgitates contained ≥2 prey types. Male and female RFBs showed no significant difference in the numerical abundance of flying fish, ballyhoo and other prey types in their regurgitates (Chi-squared test, *χ*^2^ = 0.462, *df* = 2, *p* = 0.794; Fig. 6 & Table S11). However, male BBs consumed comparatively fewer flying fish and ballyhoo, and a higher proportion of other prey, than females (Chi-squared test, *χ*^2^ = 17.896, *df* = 2, *p* < 0.001; Fig. 6 & Table S11).

**Fig. 7:**
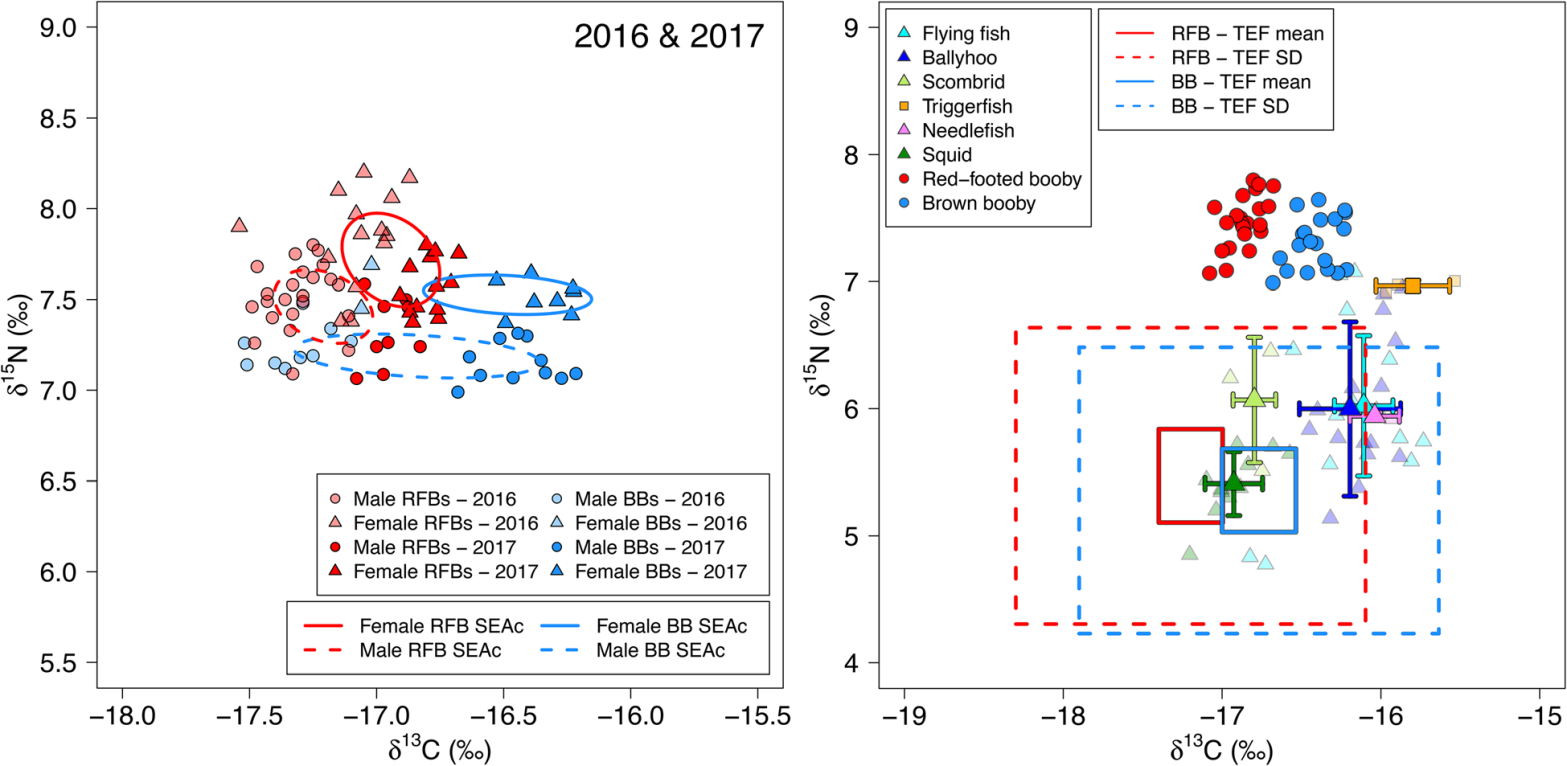
**a** Bulk stable isotope values (δ^15^N and δ^13^C) of red blood cells (RBCs) from red-footed boobies (red, *n* = 59) and brown boobies (blue, *n* = 30) according to sex (females = triangles, males = circles). Solid lines = standard ellipse areas (SEAc). **b** Booby RBC stable isotope values with respect to those of fish and squid muscle tissue sampled from regurgitates. Mean (± SD) δ^15^N and δ^13^C values of prey are shown, with raw values represented by dimmed background markers. Boxes show the expected occupied area of sampled birds in prey isotope space, using mean trophic enrichment factors of 1.96‰ ∆^15^N and 0.32‰ ∆^13^C (solid boxes), and SDs of 0.79‰ ∆^15^N and 0.86‰ ∆^13^C (dotted boxes)

RFBs were significantly more enriched in ^15^N than BBs in both sample years (GLS, *χ*^2^_4_ = 26.347, *p* < 0.001; Fig. 7a and Table 2). RFBs were also more depleted in ^13^C than BBs in 2017 (GLS, *χ*^2^_1_ = 46.047, *p* < 0.001), although no significant differences were found in ^13^C in 2016 (GLS, *χ*^2^_1_ = 0.833, *p* = 0.361; Fig. 7a & Table 2). In both species, females had higher δ^15^N (GLS, *χ*^2^_4_ = 32.647, *p* < 0.001) and δ^13^C values (GLS, *χ*^2^_1_ = 10.909, *p* < 0.001) than males (Fig. 7a), with no significant interactions between sex and year nor species detected. Despite this, a comparison of avian isotope values in both species with those of their prey showed that fractionation-corrected blood values (and their incorporated uncertainties) overlapped with the largely identical isotopic niche spaces occupied by their two main prey types (flying fish and ballyhoo; Fig. 7b).

**Table 2:** Stable isotope compositions of blood from red-footed boobies and brown boobies from the Cayman Islands

		Red-footed boobies			Brown boobies		
Isotope ratio	Year	Female	Male	All	Female	Male	All
δ^15^N (‰)	2016	7.8 ± 0.3 (14)	7.5 ± 0.2 (23)	7.6± 0.3 (37)	7.6 ± 0.2 (2)	7.2 ± 0.1 (9)	7.3 ± 0.2 (11)
	2017	7.6 ± 0.2 (13)	7.3 ± 0.2 (9)	7.5 ± 0.2 (22)	7.5 ± 0.1 (8)	7.1 ± 0.1 (11)	7.3 ± 0.2 (19)
	All	7.7 ± 0.3 (27)	7.5 ± 0.2 (32)	7.6 ± 0.3 (59)	7.5 ± 0.1 (10)	7.2 ± 0.1 (20)	7.3 ± 0.2 (30)
δ^13^C (‰)	2016	−17.1 ± 0.2	− 17.3 ± 0.1	− 17.2 ± 0.2	−17.0 ± 0.1	− 17.3 ± 0.1	−17.3 ± 0.2
	2017	−16.8 ± 0.1	− 17.0 ± 0.1	− 16.9 ± 0.1	−16.3 ± 0.1	− 16.4 ± 0.1	−16.4 ± 0.1
	All	−16.9 ± 0.2	−17.2 ± 0.2	− 17.1 ± 0.2	− 16.5 ± 0.3	− 16.9 ± 0.5	−16.7 ± 0.5

Mean (± SD) carbon and nitrogen stable isotope values for red blood cells sampled from chick-rearing red-footed boobies and brown boobies in 2016 and 2017 from populations on the Cayman Islands. Sample sizes are given in parentheses

## Discussion

This study shows that BBs and RFBs engage in different foraging behaviours - the small population of sexually-dimorphic BBs have sex-specific foraging areas close to the coast, while the larger population of weakly dimorphic RFBs travel further offshore and show almost no sex differences in foraging behaviour. These patterns can be explained by differences in dimorphism, reproductive roles, kleptoparasitism and interspecific and intraspecific competition. We discuss these potential drivers below.

### Competition and size dimorphism

Unlike some tropical seabird populations that breed throughout the year or sub-annually [61, 62], BBs and RFBs show some breeding seasonality [37, 61], resulting in potential for competition in areas of coexistence. In the Cayman Islands, the RFB population size is an order of magnitude greater than the BB population. This could lead to local prey depletion requiring RFBs to travel further from the colony, particularly during chick rearing [17, 63, 64]. This form of indirect ‘exploitative competition’ [63] may also partly explain why BBs seldom venture into coastal waters to the west of their island used by RFBs. Nevertheless, exploitative competition does not fully explain observed patterns, and segregation could arise because of species-specific differences in foraging habitat that emerge due to historical competition.

Like many tropical seabirds, BBs and RFBs both exhibit reverse sexual size-dimorphism, the former species being notably larger (this study, [37, 65]). Direct competition, whereby individuals are inhibited from access to prey by others (termed ‘interference competition’ [8]) is often attributed to body size differences [66, 67], and thought to be the main competitive force in tropical environments [68, 69]. Size differences may confer competitive advantages to BBs allowing interference with foraging opportunities for RFBs [70]. However, present day population sizes of the two species on the Cayman Islands (the BB population being small and in decline owing to anthropogenic impacts: [47, 71]) suggest that direct competition alone is unlikely to explain observed foraging differences. Furthermore, there is little evidence for intersexual competition as a driver for niche partitioning in tropical sulids [44, 72]. Rather than being driven by present day competition, the respective pelagic and coastal strategies of RFBs and BBs may instead be a ghost of competition past, or other processes that caused them to diverge.

Body size differences are also regularly suggested as an explanation for intraspecific differences in foraging behaviour [73, 74]. The relative degree of sex differences in foraging of RFBs and BBs accords with their differing degrees of dimorphism [75], as well as earlier comparisons of basic trip metrics [36, 45]. In theory, high levels of size dimorphism in BBs (23–38%, this study, [36, 76]) could allow larger females to outcompete males in colony-adjacent habitat (e.g. [73]). In comparison, the more weakly dimorphic RFBs (~ 14%, this study, [40]) exhibited almost no sex differences in foraging behaviour, which would accord with lower intraspecific competition. However, how such interference competition may operate remains unclear, although vocalisations could play a role in conveying information about size, status or sex [77, 78]. Alternatively, RFBs may have reduced scope for behavioural variation, since foraging at greater distances might cause them to experience physiological constraints on flight time, limiting scope for spatial segregation (see [79]).

### Division of labour and physiological constraints

Differing levels of sex differentiation in foraging may also relate to division of parental care [44, 80]. In both species, the larger females play greater roles in chick provision [41, 81, 82], although this division of labour is more marked in highly dimorphic BBs [41, 82]. Higher provisioning requirements may cause female BBs to remain closer to the nest, a response likely not required in RFBs that vary only slightly in their parental participation [41]. Some BB populations show an opposite pattern of foraging differentiation to those found here, with males remaining closer to shore than females [83, 84], or spending more time at the nest [35]. These cases have been attributed to selection on males to defend nest sites, and females to undertake greater roles in chick provisioning (i.e. through increased food payload capacity or more extensive travel [85, 86]). However, we propose that in the Cayman Islands ecosystem where kleptoparasitism from heterospecifics occurs (see discussion below), the need for risk aversion that likely differs with body size and sex may override relationships between payload and travel distance. Here, smaller males may undertake more distant foraging trips to minimise risks of kleptoparasitism that larger females are better able to cope with [35]. Furthermore, the longer foraging trips of males seen here, in addition to indications that male BBs have lower or similar aggressive tendencies than those of females [37, 87], suggests that territory defence may not be as biased towards males as suggested amongst sulids [35, 80, 83].

Physiological differences associated with body size and wing morphology are believed to drive resource partitioning in some seabirds (i.e. [19]). In the strongly dimorphic BBs, the smaller body size of males may confer greater aerial agility to this sex for exploiting offshore environments, where associations with conspecifics and heterospecifics likely differ from those inshore [40, 70, 88]. In comparison, in weakly dimorphic RFBs, physiological differences with sex may be less prominent. Physiological drivers could also explain interspecific differences in foraging, with smaller, more agile RFBs exploiting pelagic waters where lower wing loadings allow greater manoeuvrability during prey pursuit, which may be less important in highly coastal environments [89].

### Kleptoparasitism

Sex-based differences in kleptoparasitism may also influence observed intraspecific differences in foraging, based on the observation in 16 video-instrumented birds that all kleptoparasitic attempts were on female BBs in coastal waters. Under theories of risk aversion, the sex most vulnerable to predation pressure is predicted to minimise risk by selecting resources within safer environments [90–92]. Thus, the tendency of male brown boobies to forage further from the coast may represent risk-aversion, seeing that female frigatebirds, the only sex that we observed kleptoparasitising boobies (and a bias seen in other populations [93–95]), show a higher propensity for coastal foraging [96]. This is consistent with evidence that frigatebird density becomes more diffuse with distance from coasts [97]. Smaller, less aggressive male boobies [37, 87] may be less capable of successfully defending themselves against a challenger than females. Similarly, female frigatebirds (also the larger sex) may be more successful in, and capable of balancing the costs of, kleptoparasitism than smaller males.

Male brown boobies must still travel through coastal waters in which kleptoparasites predominantly operate to reach foraging sites, suggesting that they do encounter frigatebirds. However, all kleptoparasitic interactions occurred during or closely timed with booby foraging activity (Fig. S9). This foraging-related context of piracy may allow transiting males to avoid regular kleptoparasitism, while short-ranging foraging females experience higher exposure. Frigatebirds are known to wait aloft near colonies to attack boobies as they return from foraging trips ladened with food [94, 98]. However, in our study system, brown booby nests are scattered along large stretches of coast, with no defined travel corridor or focal point to target. Therefore, use of a ‘waiting tactic’ is unlikely to yield higher benefits for kleptoparasites over one where frigatebirds target foraging individuals or feeding aggregations.

This mechanism could also help to explain the observed interspecific differences in behaviour. While we could not equip RFBs with video loggers, casual observations at or near nesting sites suggest that rates of kleptoparasitism in coastal waters near colonies may be higher on smaller-bodied RFBs than larger BBs (Austin et al. unpublished observation), the former of which nest side-by-side with magnificent frigatebirds on Little Cayman [99]. Frigatebirds congregate in large groups near the RFB colony and regularly partake in kleptoparasitic attempts on RFBs as they return from foraging trips, as seen in other co-existing populations [93, 100, 101]. This stressor is likely to influence foraging behaviour (see also [98]), and may drive a pelagic avoidance tactic in both sexes of RFBs. Kleptoparasitism might also explain differences in diel activity patterns of the two species: RFBs predominantly leave and return to the colony in crepuscular hours or under cover of darkness (Additional file 1 and see [98]), while BBs show more variability in departure and return times, which largely occur during daylight (Additional file 1). Nevertheless, the role of kleptoparasitism in shaping behaviour of the two species warrants further investigation.

### Dietary partitioning

Partitioning in diet can alleviate competitive pressures in communities [10, 102], but we found weak evidence for this in our dietary data with both species targeting similar prey (see also [30, 72, 103, 104]). In accordance with their neritic distribution, there was a higher diversity of prey in regurgitates of coastal BBs, including reef-associating species, and a higher incidence of squid in pelagic RFBs (consistent with [105, 106]). While no sex differences were found in the diet of RFBs, there were differences in the relative contribution of different prey in female and male BBs, likely relating to sex differences in habitat use. Nevertheless, both species predominantly targeted flying fish and ballyhoo that occupy similar ecological niches [107].

Stable isotope values of both species fell within similar isotope prey space seen in our reference data, further indicating that the two populations do not substantially differ in their dietary resources. This broad similarity in diet likely reflects the flexible and opportunistic foraging strategies required in oligotrophic tropical environments where prey are widely distributed [24, 103]. Thus, it is unlikely that differences in habitat use are driven by exploitation of differing target prey. Differences between isotopic values of RFBs and BBs are consistent with commonly observed inshore-offshore gradients in food web isotopes [108, 109], with pelagic RFBs being more enriched in ^15^N than coastal feeding BBs across sampling years, and more depleted in ^13^C in 2017. Overarching between-year differences in both species most likely reflect variability in oceanographic conditions and associated biogeochemical processes. In both species, females had higher δ^15^N and δ^13^C values than males. While this pattern may be explained in BBs by the tendency of females to stay closer to the coast, RFBs did not show significant differences in space use with sex. Nevertheless, the larger size of females may allow exploitation of larger prey, which could be reflected in nitrogen isotope values. Small sample sizes prevented a comparison of prey size between sexes, but evidence in tropical seabirds of a strong correlation between body mass and prey length [30] supports this suggestion. Alternatively, overriding sex differences may be associated with reproductive processes such as egg synthesis, should fluctuations in isotopic routing and fractionation span multiple months for RBCs [110]. While there was little evidence for a role of diet in driving foraging differences in the two focal sulids, differing nutritional requirements could still influence use of habitats and foraging strategies, as is now being discussed and tested in seabirds [111, 112].

The fact that little inter- and intraspecific segregation in dive behaviour was found, with the exception of slightly greater dive depths in BBs (which can be explained by body mass differences or consumption of reef-associating prey), further supports the conclusion that these two seabirds have not evolved vastly different dietary niches, and are likely constrained in the diversity of prey that they can access within tropical surface waters [30].

## Conclusions

An improved understanding of foraging diversification between coexisting species in tropical environments may help to predict how future change in marine environments may impact species distributions and the functioning of communities, and thus their vulnerability to environmental perturbation. For example, should coastal habitats in the study system offer more predictable resources than those offshore, BBs and RFBs may show differing levels of specialism and differing adaptive capacities to prey field lability (e.g. [113]). Devising explicit tests of the mechanisms underlying foraging segregation in natural systems remains challenging, but our data suggest that a combination of factors linked to population size and body size may contribute, including division of labour, exploitative competition and kleptoparasitism. This is supported by evidence of local adaptation in both species indicated through a range of intraspecific behavioural patterns reported amongst populations [35, 36, 42, 43, 83, 84, 114]. This highlights the need for further comparative studies within and across a range of marine environments, including within the tropics, to improve knowledge of processes acting on seabird community structure and the vulnerability of constituent species to environmental change*.*

## Supplementary Information

**Additional file 1.** Supporting materials. Supporting materials in the form of figures, tables and text compiled in a single document.

## Data Availability

The datasets supporting the conclusions of this article are available upon request from the corresponding author.
