# Supplementary material for: Interspecific and intraspecific foraging differentiation of neighbouring tropical seabirds
Source: Mov Ecol. 2021 May 26;9:27. doi: 10.1186/s40462-021-00251-z (PMC8152358; doi:10.1186/s40462-021-00251-z)
Supplement: Supplementary file 2 — Additional file 1. Supporting materials. Supporting materials in the form of figures, tables and text compiled in a single document. [file 40462_2021_251_MOESM1_ESM.docx]

*This additional file accompanies the article:*

**Interspecific and intraspecific foraging differentiation of neighbouring tropical seabirds**

Austin R.E.*^1^, De Pascalis F.^1,2^, Votier S.C.^3^, Haakonsson J.^4^, Arnould J.P.Y.^5^, Ebanks-Petrie G.^4^, Newton J.^6^, Harvey J.^4,7^ & Green J.A.^1^

^1^School of Environmental Sciences, University of Liverpool, Liverpool, L69 3GP, UK

^2^Current institution: Department of Environmental Science and Policy, University of Milan, Milan, Italy

^3^The Lyell Centre, Heriot-Watt University, Edinburgh, EH14 4AP, UK

^4^Department of Environment, Cayman Islands Government, Grand Cayman, KY1-1002, Cayman Islands

^5^School of Life and Environmental Sciences, Deakin University, Burwood, VIC 3125, Australia

^6^NERC National Environmental Isotope Facility, Scottish Universities Environmental Research Centre, Scottish Enterprise Technology Park, East Kilbride, G75 0QF, UK

^7^Current institution: Guy Harvey Ocean Foundation, Grand Cayman, KY1-1005, Cayman Islands

*Corresponding author: [rhiannoneaustin@gmail.com](mailto:rhiannoneaustin@gmail.com), [R.E.Austin@liverpool.ac.uk](mailto:R.E.Austin@liverpool.ac.uk)

**Appendix S1 – Chick age range**

**Table S1.** Number of tracked breeding red-footed boobies and brown boobies with respect to chick age.

| **Species** | **Small chicks**  **(1-3 wks)** | **Medium chicks**  **(4-8 wks)** | **Large chicks**  **(9-13 wks)** |
| --- | --- | --- | --- |
| Red-footed booby | 2 (0.08) | 21 (0.88) | 1 (0.04) |
| Brown booby* | 15 (0.26) | 33 (0.57) | 10 (0.17) |

*****No evidence to suggest that chick age influenced trip duration significantly within the range of ages sampled in the study (GLMM with a random individual intercept, LRT, 𝝌^2^_2_ = 1.099, *p* = 0.147). Formal statistical testing not possible with red-footed boobies owning to small sample sizes in small and large chick categories.

**Appendix S2 – Comparison of interpolated ‘original’ and ‘down-sampled’ GPS tracks**

**
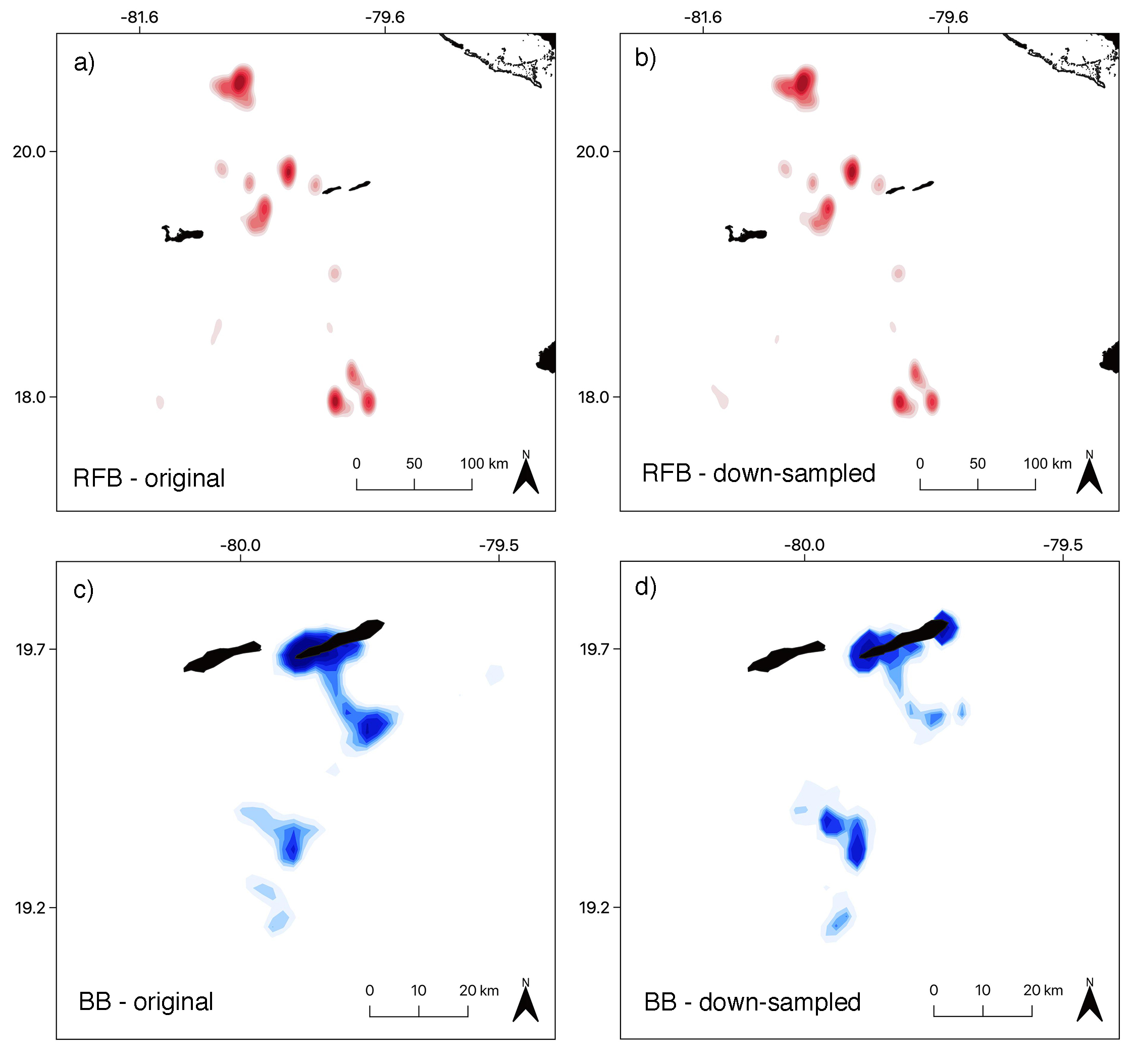
**

**Fig S1.** Comparison of Kernel Density Estimates of foraging locations from a-b) red-footed boobies (*n* = 3) and c-d) brown boobies (*n* = 3) for the same foraging trips processed at different sampling rates prior to curvilinear interpolation. Original = 30 s interpolated GPS locations recorded at ~30-40 s intervals prior to interpolation. Down-sampled = 30 s interpolated GPS locations down-sampled to ~2 min intervals from ‘original’ data prior to interpolation.

For both species, the majority of foraging tracks were recorded at ~30-40 s resolution (RFBs = 92% of birds, BB = 60% of birds). However, to investigate whether inclusion of 2 min tracks influenced the resulting Kernel Density Estimates (KDE) of foraging trips, we undertook the following analysis: Three tracks for each species recorded at ~30-40 s resolution were randomly selected. We then compiled two sets of data for each individual prior to interpolating the data to the same time intervals: 1) ‘original’ data recorded at ~30-40 s intervals and 2) ‘down-sampled’ versions of ‘original’ data that had been sub-sampled to ~2 minute intervals. Both sets of data were then interpolated to 30 s intervals, and behavioural states were estimated for locations using 3-state Hidden Markov Models following the method described for the full GPS dataset in the main text. For each species, locations estimated to be associated with foraging were then used to create KDEs for the two datasets. Bhattacharyya’s affinity was calculated as a measure of overlap between 50% and 90% kernel contours, and for both species there was little difference in the resulting KDEs (BA overlap: RFBs, 50% KDE contour = 99%, 90% contour = 99%; BBs, 50% contour = 67%, 90% contour = 85%). Thus, we concluded that inclusion of the different sampling rates did not result in misleading interpretations about levels of segregation and space use between our two study species (Fig S1).

**Appendix S3 -** **Discriminant Function Analysis**

We trained a Discriminant Function Analysis (DFA) on morphometric measurements from RFB individuals of known sex (classified using DNA sexing; *n* = 69), in order to predict the sex of RFBs that blood or feather tissue were not collected from. The morphometric measurements used in the DFA were chosen from a pool of candidates (mass, wing length, bill length, bill depth, bill width, tail length, tarsus length) using a Wilks Lambda stepwise forward variable selection method. The variables that contributed significantly to separating the two sexes were body mass, wing length and bill length. The DFA on training data (containing birds of known sex) correctly assigned 94% of individuals to their true sex (Wilks’ Lambda = 0.357, *F* = 39.07, P < 0.001; Table S2). Similarly, a leave-one-out cross validation on the data had 93% accuracy. The discriminant function obtained from this analysis was then used to predict the sex of 10 unsampled birds (3 of which had matching spatial data), and the assignments were used in subsequent analyses. Fisher’s classification coefficients for the two sexes are given in equations 1 and 2.

**Table S2.** Outputs of a Discriminant Function Analysis on training data using morphometric measurements (wing length, bill length and body mass) of red-footed boobies of known sex. Proportions of males and females that were accurately assigned to their true sex are shown. Numbers in brackets show frequencies.

|  |  | Predicted | |
| --- | --- | --- | --- |
|  |  | Female | Male |
| Actual | Female | 0.96 (27) | 0.04 (1) |
|  | Male | 0.07 (3) | 0.93 (38) |

**Fisher’s classification coefficients:**

Equation S1.

*D*_Female_ = 0.043 * Mass + 4.502 * WingLength +0.124 * BillLength – 1455.604

Equation S2.

*D*_Male_ = 0.008 * Mass + 4.379 * WingLength + 12.046 * BillLength – 1345.112

**Table S3.** Summary of the morphometric characteristics (mean ± SD) of adult red-footed boobies (*n*, F = 28, M = 41) and brown boobies (*n*, F = 25, M = 33) from breeding populations on the Cayman Islands. The mean percent difference between metrics for females and males, and effect sizes (Cohen’s *d*) for species (*d_spp_*) and sex (*d_sex_*) comparisons are shown. All measurements except body mass are given in mm. Parameters from generalised least squares (GLS) models and linear models to compare between species and sex are shown.

| Species | Sex | Mass (g)** | Tarsus length* | Wing length* | Bill length* | Tail length |
| --- | --- | --- | --- | --- | --- | --- |
| Red-footed booby | Female | 914 ±49 | 36.3 ±2.4 | 395 ±10 | 87.9 ±2.8 | 231 ±18 |
|  | Male | 795 ±55 | 35.0 ±1.9 | 380 ±9 | 84.6 ±2.4 | 232 ±15 |
|  | **All** | **843 ±78.5** | **35.5 ±2.2** | **386 ±12** | **85.9 ±3.0** | **231 ±16** |
|  | % size diff. | 15.0 | 3.7 | 4.0 | 3.9 | 0.4 |
|  | *d_sex_* | 2.3 (1.6 – 2.9) | 0.7 (0.2 – 1.2) | 1.6 (1.1 – 2.2) | 2.4 (2.0 – 2.9) | -0.1 (-0.5 – 0.4) |
| Brown booby | Female | 1213 ±96 | 47.6 ±4.4 | 417 ±7 | 100.7 ±8.1 | 200 ±11 |
|  | Male | 982 ±98 | 46.1 ±3.3 | 397 ±10 | 96.2 ±4.7 | 201 ±10 |
|  | **All** | **1081 ±151** | **46.8 ±3.8** | **406 ±13** | **98.2 ±6.7** | **201 ±10** |
|  | % size diff. | 23.5 | 3.3 | 5.0 | 4.7 | 0.5 |
|  | *d_sex_* | 2.4 (1.7 – 3.1) | 0.4 (-0.2 – 0.9) | 2.3 (1.6 – 3.0) | 0.7 (0.2 – 1.2) | -0.1 (-0.6 – 0.4) |
|  | *d_spp_* | 2.0 (1.6 – 2.5) | 3.7 (3.1 – 4.3) | 1.6 (1.2 – 2.0) | 1.3 (0.8 – 1.8) | -2.2 (-2.6 - -1.7) |
| LRT (*p*) | Species | 93.75 (<0.001) | 131.30 (<0.001) | 96.76 (<0.001) | 152.32 (<0.001) | 96.73 (<0.001) |
|  | Sex | 99.78 (<0.001) | 9.53 (0.002) | 82.49 (<0.001) | 12.57 (0.002) | 0.169 (0.681) |
|  | Sex:Species | 224.95 (<0.001) | --- | --- | --- | --- |

*****Models with terms for Species, Sex, **Model with terms for Species, Sex and Species:Sex. A correlation structure to allow unequal variances for species was used for tarsus, mass and tail length models, and correlation structures for both species and sex were used for bill length (GLS models). No correlation structure was needed for wing length (linear model).

**Appendix S4 - Behavioural classification in booby foraging trips**

Hidden Markov Models, based on correlated step lengths and turning angles, were fit to estimate behaviour in foraging tracks of RFBs and BBs and identify foraging bouts for use in further analyses (R package ‘momentuHMM’). Model-estimated states were validated using dive and immersion data from a subset of individuals simultaneously tracked with TDR (dive activity) and GLS (immersion patters) loggers. Following a comparison of the negative log-likelihood values of candidate models with between 2 and 5 states (see model selection methods outlined in Dean et al 2013), HMMs with 3 hidden states were used to estimate behaviour.

**Table S4.** Mean (± SD) step lengths, and concentrations of turn angles, for the three states classified by HMMs for GPS-tracked red-footed boobies and brown boobies.

| HMM state | Red-footed booby | | Brown booby | |
| --- | --- | --- | --- | --- |
|  | Step length (km) | TA Conc. | Step length (km) | TA Conc. |
| Travel - 1 | 0.30 ± 0.06 | 38.03 | 0.30 ± 0.09 | 38.16 |
| Forage - 2 | 0.08 ± 0.09 | 1.19 | 0.11 ± 0.08 | 1.68 |
| Rest - 3 | 0.01 ± <0.01 | 14.06 | 0.01 ± 0.01 | 0.72 |

| State at *t* | RFB | 1 | 2 | 3 |
| --- | --- | --- | --- | --- |
|  | 1 | 0.963 | 0.037 | <0.001 |
|  | 2 | 0.024 | 0.931 | 0.045 |
|  | 3 | <0.001 | 0.057 | 0.944 |

| State at *t* | BB | 1 | 2 | 3 |
| --- | --- | --- | --- | --- |
|  | 1 | 0.937 | 0.063 | <0.001 |
|  | 2 | 0.050 | 0.912 | 0.038 |
|  | 3 | <0.001 | 0.044 | 0.955 |

**Table S5.** State transition matrix from the three-state Hidden Markov Model, showing the probability of changing from state at time *t* to state at time *t* +1. State, 1 = travel, 2 = forage, 3 = rest. Proportion of locations assigned to states: RFB, 1 = 0.27, 2 = 0.41, 3 = 0.32; BB, 1 = 0.31, 2 = 0.38, 3 = 0.32.


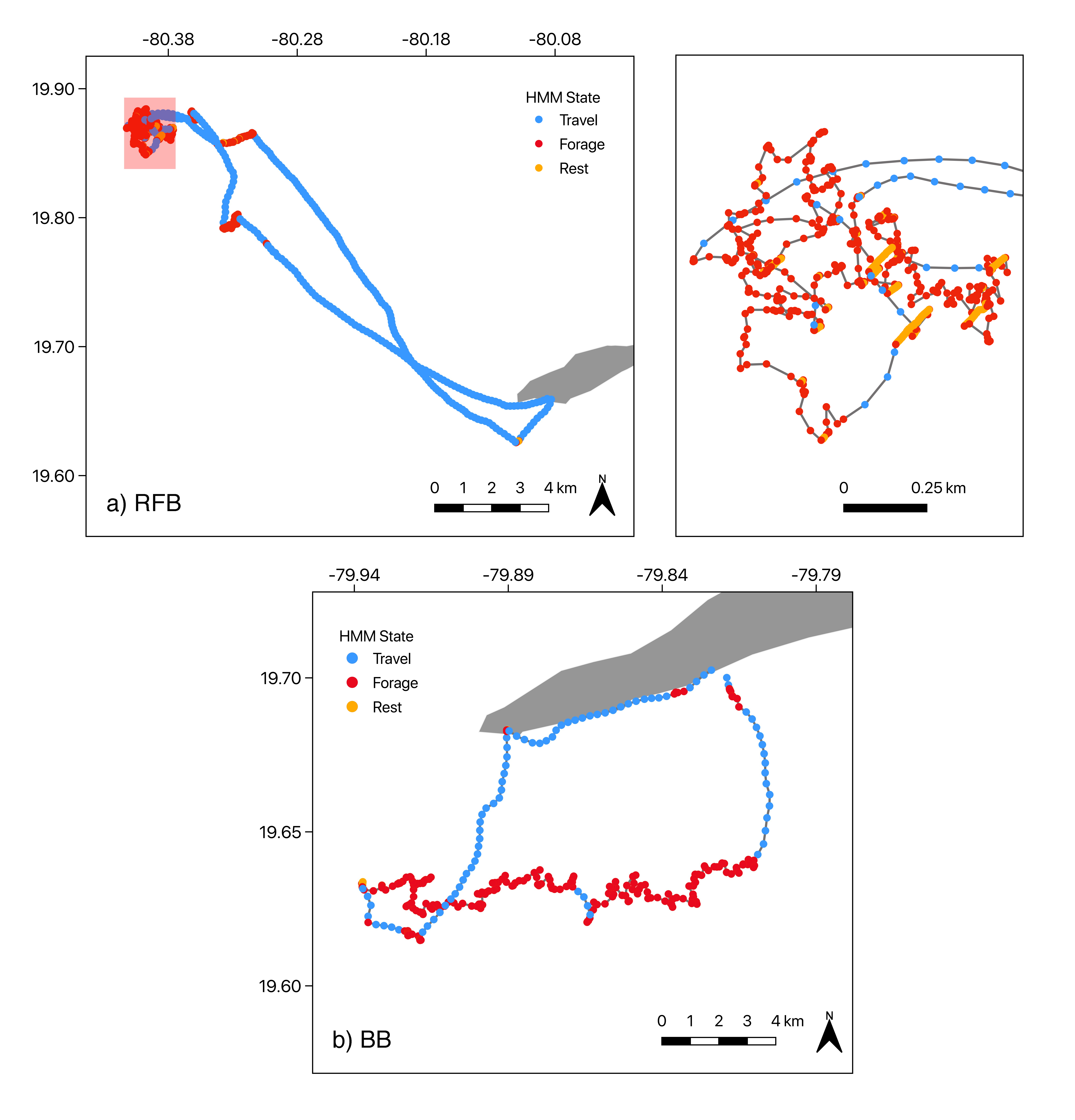


**Fig. S2** Example tracks from a a) red-footed booby (RFB) and b) brown booby (BB), coloured according to behavioural states estimated from 3-state HMMs. The pink box in the left RFB figure highlights the position of the zoomed in figure on the right.

**Table S6**. The proportion of GPS locations associated with dive activity (Dives*_p_*; *n* birds, RFBs = 8, BBs = 18), and the mean (± SD) proportion of time spent on-water in 30 s sections of GPS tracks (OW*_t_*; *n* birds, RFBs = 10, BBs = 13), for the three behavioural states estimated by Hidden Markov Models (HMM).

|  | RFB | | BB | |
| --- | --- | --- | --- | --- |
| State | Dives*_p_* | OW*_t_* | Dives*_p_* | OW*_t_* |
| Travel | 0.13 | 0.01±0.02 | 0.15 | 0.02±0.02 |
| Forage | 0.77 | 0.39±0.14 | 0.80 | 0.17±0.11 |
| Rest | 0.10 | 0.98±0.03 | 0.05 | 0.44±0.44 |


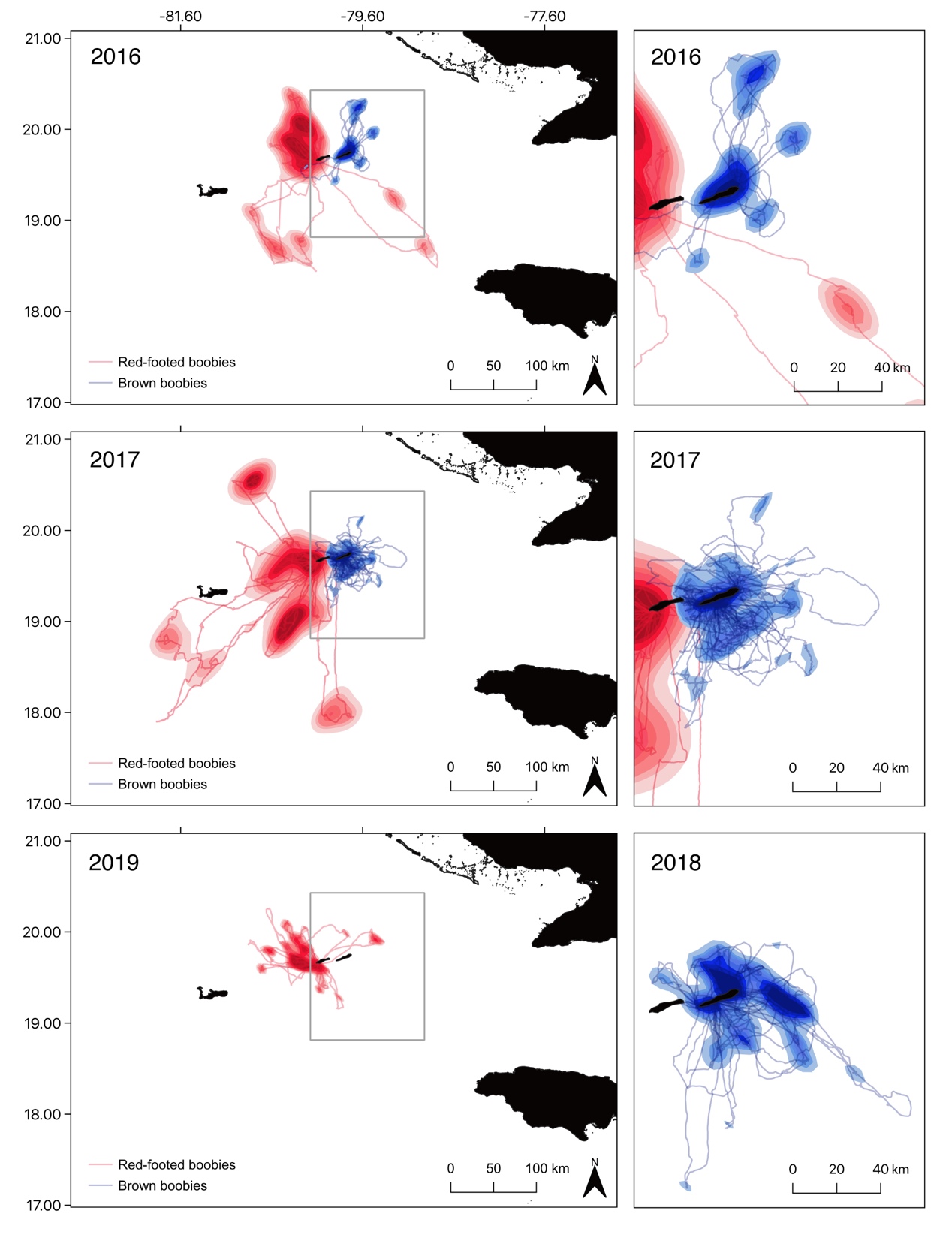


**Fig. S3** Foraging tracks and Kernel Density Estimates of red-footed boobies and brown boobies, tracked with GPS from neighbouring populations in the Cayman Islands during breeding seasons between 2016 and 2019. Data are presented separately for each tracking year. Only BBs were tracked in 2018 and only RFBs in 2019 (bottom row).

**Appendix S5 - Trip clustering**

A PCA was run on rescaled trip characteristics to identify collinearity and redundancy, and extract appropriate variables for further clustering of foraging trips. All variables contributed relatively equal contributions to the first principle component (PC1), with the exception of ‘mean underlying bathymetry’, which also contributed little to the second principle component (PC2). Trip duration contributed >42% to PC2 (Fig S4). Trip duration (TripDuration_hr), mean distance to nearest coastline (meanNDist) and maximum distance (Maxdist) were chosen for use in subsequent analysis. While trip duration and maximum distance were correlated, incorporation of both variables improved the clustering and allowed us to mitigate potential errors introduced by averaging ‘distance to nearest coastline’ within tracks.

**Fig S4.** PCA biplot showing the variables used in the analysis (indicated with arrows and labels) and position of individual data points coloured according to species (BB = red, RFB = blue). 95% ellipses are given for the two species. Arrow lengths from the origin indicate the quality of each variable on the map and representation on the principle components.

**
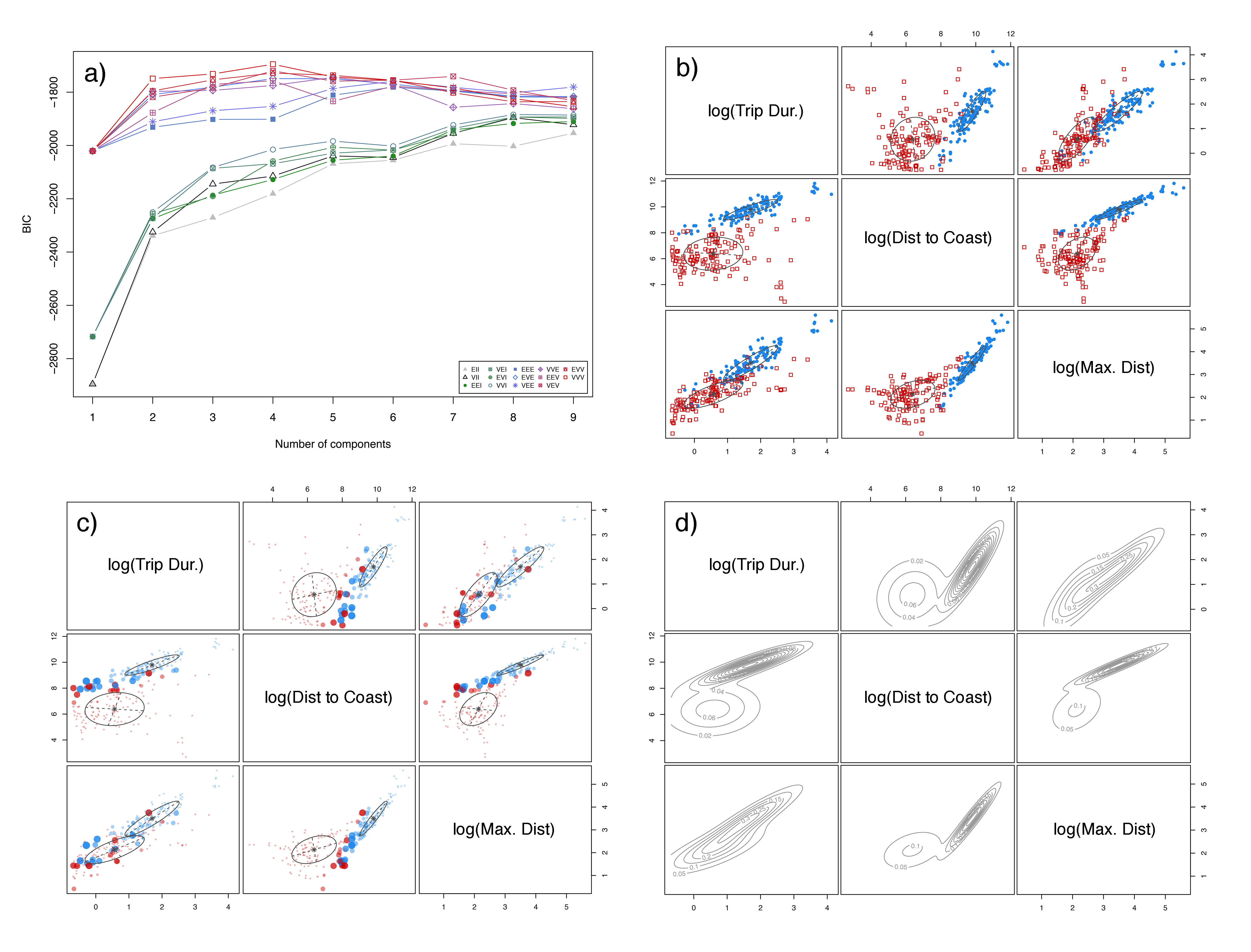
Fig. S5** Results from Gaussian Mixture Model (GMM) selection procedures, and outputs of the final two-state model. a) Bayesian Information Criteria (BIC) for candidate GMMs with 1 to 9 components, b) GMM-assigned clusters based on ‘trip duration’, ‘mean distance to nearest coastline’ and ‘maximum distance’, c) uncertainty of belonging to one of the two GMM-assigned clusters and d) estimated density based on ‘trip duration’, ‘mean distance to nearest coastline’ and ‘maximum distance’. State: blue = 1, red = 2.

**Appendix S6 – Isotopic discrimination**

**Table S7** Published carbon and nitrogen stable isotope discrimination factors between seabird blood to prey muscle tissue.

| **Seabirds** | **Diet** | **Diet tissue** | **Avian tissue** | **Condition** | ***n*** | Δ**15N** | Δ**13C** | **Source** |
| --- | --- | --- | --- | --- | --- | --- | --- | --- |
| Ring-billed gull (Larus delawarensis) | Perch† | Whole* | Whole blood | Captive | 14 | 3.1±0.2 | 0.3±0.8 | Hobson & Clark, 1992 |
| **Greak skua (*Catharacta skua*)** | **Sprat** | **Whole*** | **Whole blood** | **Captive** | **9** | **2.8** | **1.1** | **Bearhop et al., 2002** |
|  | Beef | NA | Whole blood | Captive | 9 | 4.2 | 2.3 | Bearhop et al., 2002 |
| **King penguin (*Aptenodytes patagonicus*)** | **Herring** | **Muscle*** | **Whole blood** | **Captive** | **10** | **1.23** | **-0.61** | **Cherel et al., 2005** |
|  |  | Whole* | Whole blood | Captive | 10 | 2.07 | -0.81 | Cherel et al., 2005 |
| **Rockhopper penguin (*Eudyptes chrysocome*)** | **Capelin** | **Muscle*** | **Whole blood** | **Captive** | **9** | **1.86** | **0.46** | **Cherel et al., 2005** |
|  |  | Whole* | Whole blood | Captive | 9 | 2.72 | 0.02 | Cherel et al., 2005 |
| Rhinoceros auklet (*Cerorhinca monocerata*) †† | Silverside | Whole | Red blood cells | Captive | 18 | 2.84-3.49 |  | Sears et al., 2009 |
| **Mean ± SD (lipid extracted, marine diet, adults)^** | |  |  |  |  | **1.96 (±0.79)** | **0.32 (±0.86)** |  |
| *Lipid extracted tissue; †also fed vitamin supplements; ††blood sampled from chicks & juveniles.  ^Mean ± SD calculated from studies with lipid-corrected carbon isotope values from adults fed on a marine diet | | | | |  |  |  |  |

**Appendix S7 – Breeding success**

**Table S8** Fledgling success (number of eggs that hatched and fledged / total number of eggs laid) of experimental and control nests for red-footed boobies and brown boobies from colonies on the Cayman Islands. Sample sizes are given in brackets. Bold treatments in each column are those used for statistical comparisons.

|  | Brown boobies | | | Red-footed boobies | | |
| --- | --- | --- | --- | --- | --- | --- |
| Treatment | 2016 | 2017^†^ | 2018^†^ | 2016 | 2017 | 2019 |
| Control | **0.93 (43/46)** | **1.00 (13/13)** | **0.79 (15/21)** | **0.78 (32/41)** | **0.32 (52/163)** | **---** |
| Experimental | --- | **0.96 (27/28)** | **---** | --- | **0.71 (24/34)** | **1.00 (24/24)** |
| *GPS* | **1.00 (11/11)** | 1.00 (11/11) | --- | **0.82 (23/28)** | 0.82 (9/11) | 1.00 (14/14) |
| *Combined* | --- | 0.94 (16/17) | **0.95 (19/20)^^^** | --- | 0.65 (15/23) | 1.00 (10/10 |
| *P* | 1.000 | 1.000 | 0.093 | 0.767 | <0.001 | --- |
| *Power* | 0.06 | 0.07 | 0.07 | 0.89 | 0.99 | --- |

^†^Control group consisted of birds monitored over the same time period as tracked nests; ^Fledging data not collected for one study nest in 2018.

**Appendix S8 – Tracking**

**Table S9** Summary of tracked red-footed boobies and brown boobies during the study. The number of working loggers recovered is given in brackets.

|  | Brown boobies | | | Red-footed boobies | | |
| --- | --- | --- | --- | --- | --- | --- |
| Tags | 2016 | 2017 | 2018 | 2016 | 2017 | 2019 |
| GPS | 13 (8) | 36 (32) | 36 (28) | 20 (11) | 36 (13) | 24 (7) |
| *TDR* | --- | 18 (15) | 12 (12) | --- | 27 (18) | 10 (2) |
| *GLS* | --- | 18 (15) | --- | --- | 24 (17) | --- |


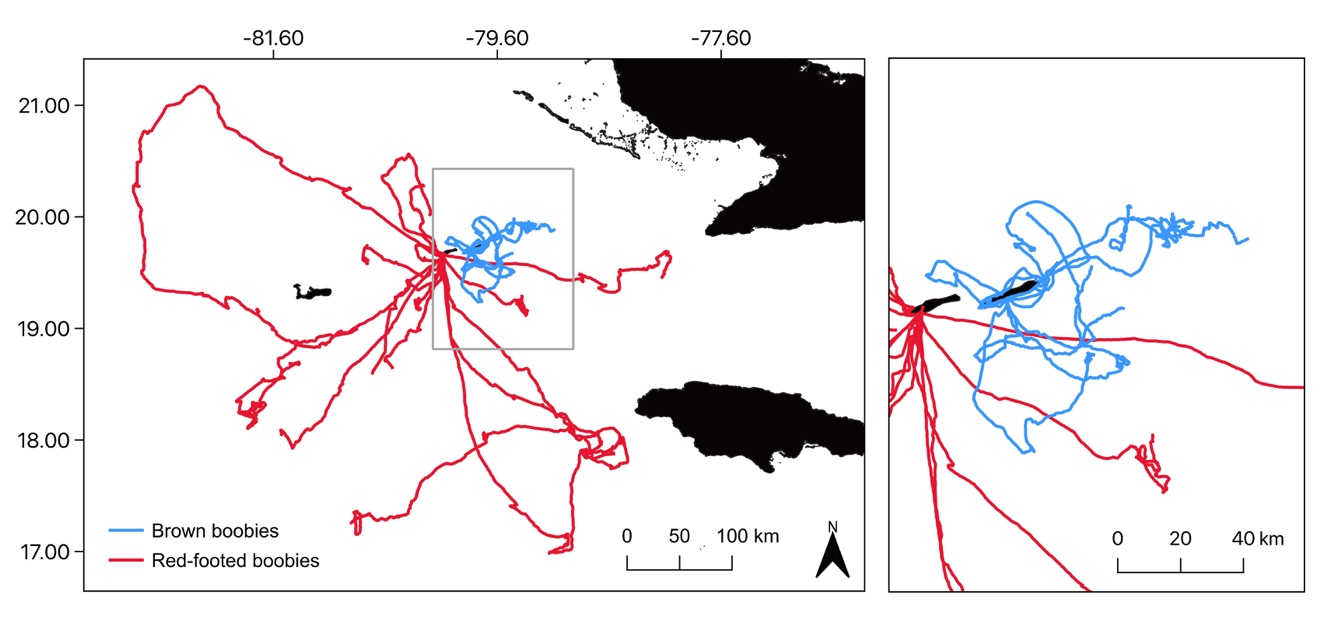


**Fig. S6** Partial foraging trips for red-footed boobies (red; *n* = 14) and brown boobies (blue; *n* = 13) tracked with GPS loggers between 2016 and 2019.


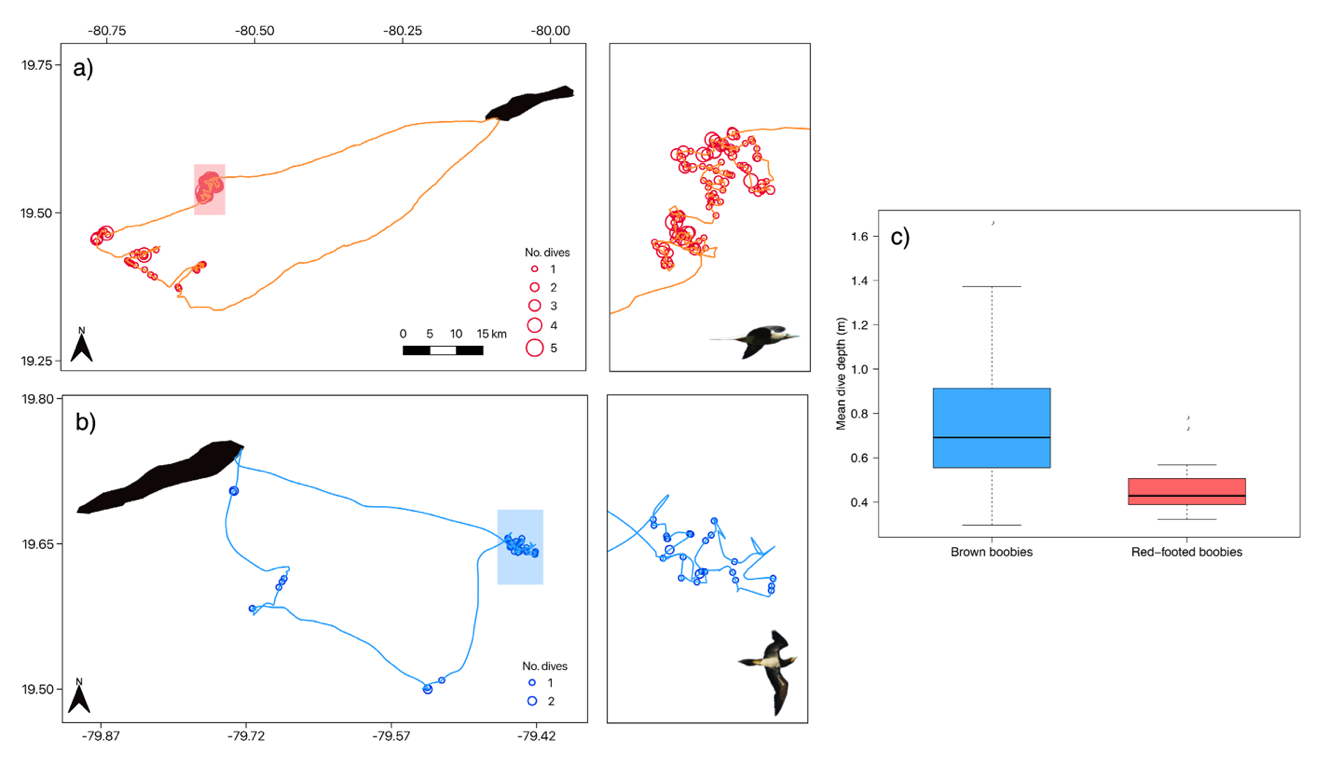


**Fig. S7** Example tracks of a) red-footed boobies and b) brown boobies, showing the spatial distribution and number of dives (circles), and c) the distribution of maximum dive depths for the two species (red = red-footed boobies, *n* = 8; blue = brown boobies, *n* = 18).


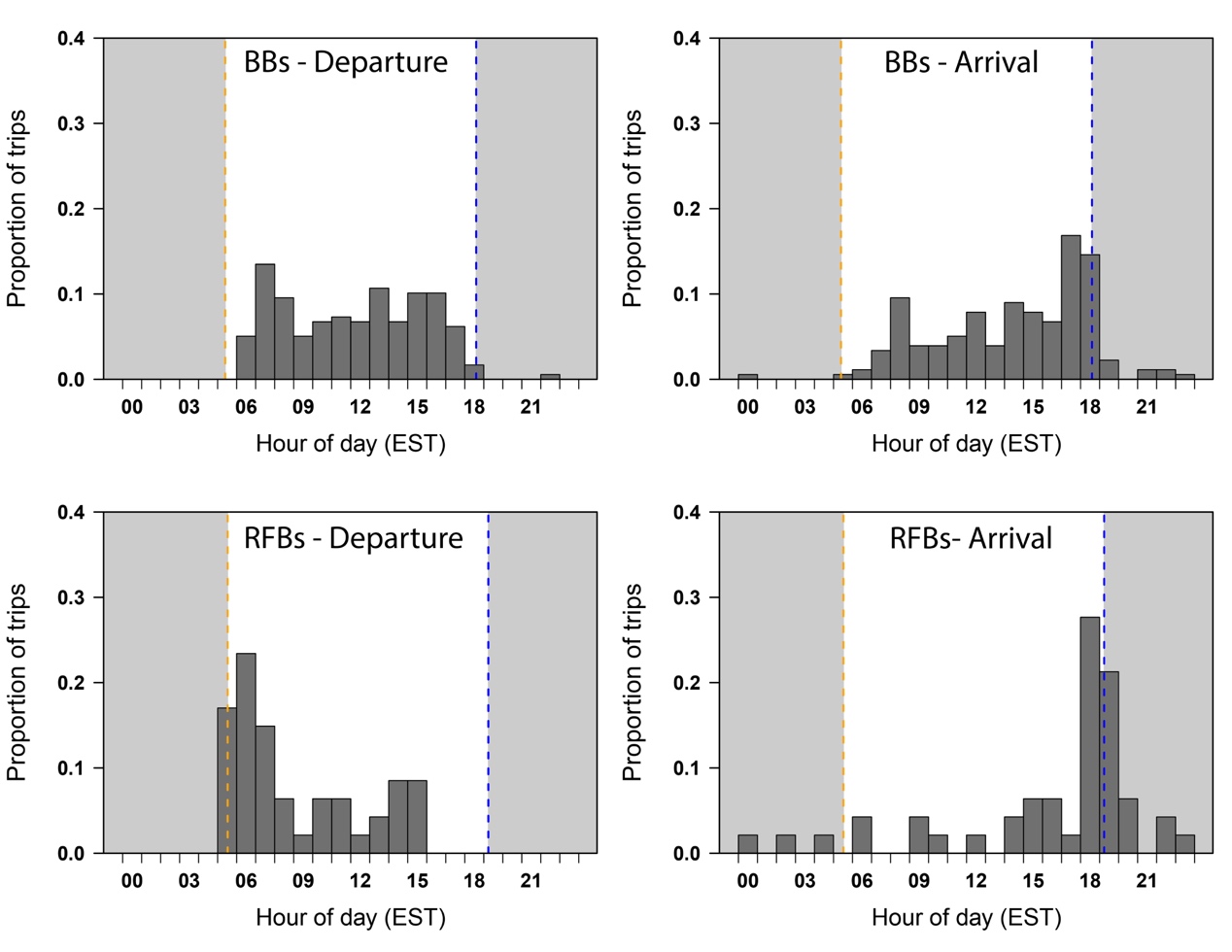


**Fig. S8** Departure and arrival times of foraging trips from breeding red-footed boobies and brown boobies, tracked with GPS loggers from populations on the Cayman Islands between 2016 and 2019. Dotted lines show average dawn (orange) and dusk (blue) times over tracking periods.

**Appendix S9 – Kleptoparasitism**

**Table S10.** Summary of kleptoparasitic interactions between magnificent frigatebirds (FB) and video-instrumented brown boobies from the Cayman Islands.

| Attempt | Date | Bird ID | Start time | Dur (s) | FB Life Stage | No. FBs | Evasive Landing | Victim | Body Contact | Coast dist. (km) |
| --- | --- | --- | --- | --- | --- | --- | --- | --- | --- | --- |
| 1 | 19/02/2018 | 1 | 10:34:00 | 31 | I | 1 | Y | F | N | NA |
| 2 | 19/02/2018 | 1 | 13:20:36 | 7 | A | 2 | Y | F | N | NA |
| 3 | 20/02/2018 | 2 | 09:33:26 | 14 | A | 1 | N | C/F | Y - wing | 1.5 |
| 4 | 17/02/2018 | 3 | 11:36:35 | 8 | I | 1-2 | Y | F | Unknown | 12.9 |
| 5 | 23/02/2018 | 4 | 12:00:43 | 32 | A | 1 | Y | F | N | 0.4 |
| 6 | 23/02/2018 | 4 | 12:08:20 | 30 | A | 3 | Y | F | Unknown | 0.5 |
| 7 | 23/02/2018 | 4 | 12:10:39 | 6 | A | 1 | Y | F | Unknown | 0.5 |
| 8 | 23/02/2018 | 4 | 12:12:28 | 45 | A | 1 | Y | F | Y - bill/wing | 0.5 |
| 9 | 23/02/2018 | 4 | 12:14:28 | 6 | A | 1 | Y | F | N | 0.5 |
| 10 | 23/02/2018 | 4 | 14:11:26 | 4 | A | 1 | Y | F | N | 0.5 |
| 11 | 22/02/2018 | 5 | 17:51:14 | 36 | A | 1 | Y | F | Unknown | 0.8 |
| 12 | 22/02/2018 | 5 | 17:52:43 | 4 | A | 1 | Y | F | N | 0.9 |

A = adult, I = immature; C = conspecific, F = focal booby, Dur = duration, Coast dist . = distance to nearest coastline.

**
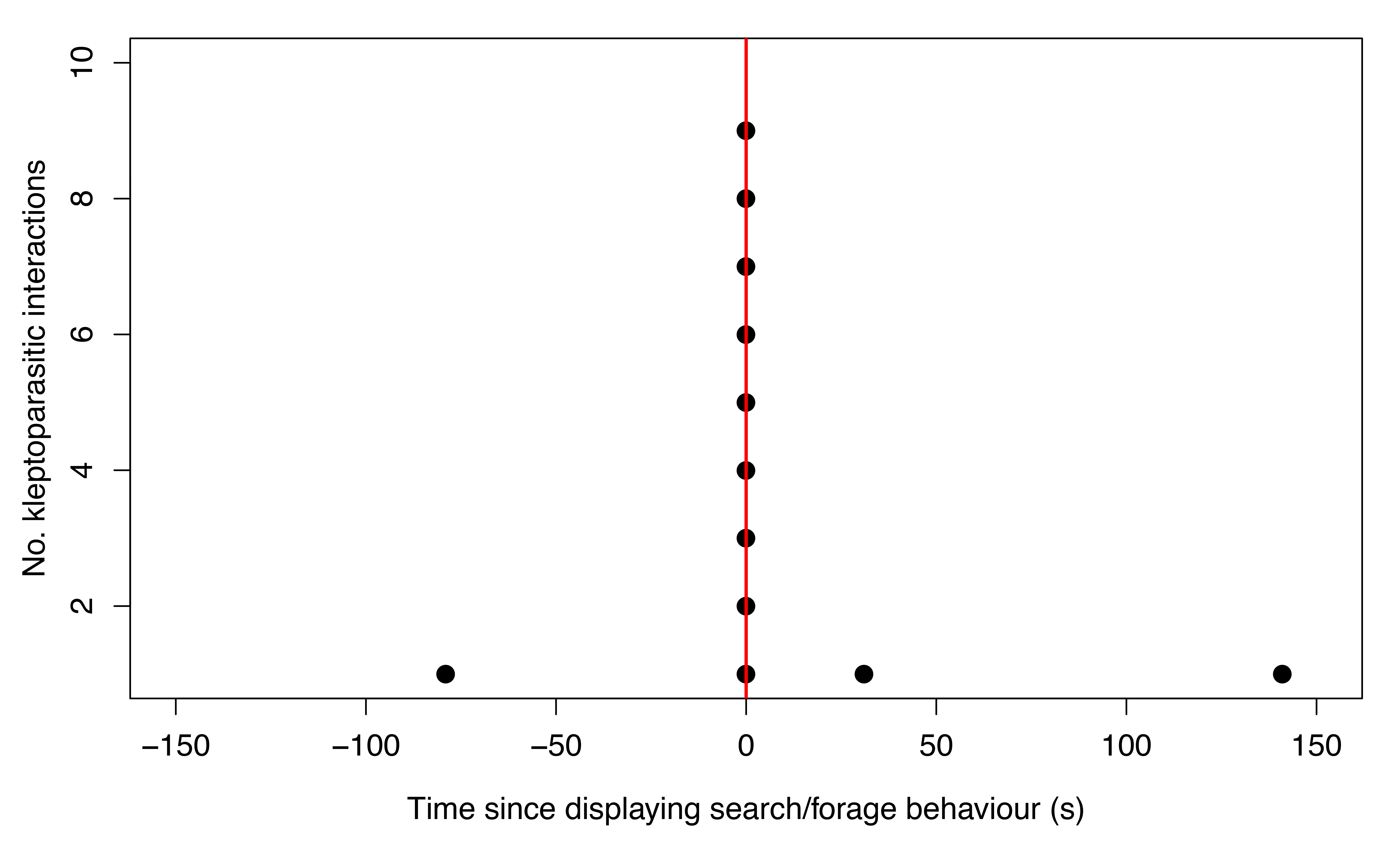
**

**Fig. S9** Time that video-instrumented brown boobies were assessed as engaging in search or foraging behaviour relative to the start of kleptoparasitic interactions with magnificent frigatebirds (red line; *n* = 12).

**Appendix S10 - Summary of diet**

**Table S11** Contribution of different prey types to the diet of red-footed boobies (*n* = 15) and brown boobies (*n* = 30) as found in regurgitate samples collected between 2016 and 2018. The numerical abundance (N%; the percentage of a prey type out of all prey sampled) and frequency of occurrence (O%; the percentage of birds with a prey type present in their regurgitate) of each prey type is given.

| Prey type* | Red-footed boobies | | | | | | Brown boobies | | | | | |
| --- | --- | --- | --- | --- | --- | --- | --- | --- | --- | --- | --- | --- |
|  | ♀ (*n* = 6) | | ♂ (*n* = 6) | | All (*n* = 15) | | ♀ (*n* = 18) | | ♂ (*n* = 9) | | All (*n* = 30) | |
|  | N% | O% | N% | O% | N% | O% | N% | O% | N% | O% | N% | O% |
| BH | 13 | 17 | 12 | 17 | 11 | 13 | 43 | 72 | 14 | 44 | 33 | 63 |
| FF | 54 | 67 | 65 | 83 | 60 | 73 | 24 | 56 | 12 | 56 | 20 | 53 |
| DF | 0 | 0 | 0 | 0 | 0 | 0 | 0 | 0 | 2 | 11 | 1 | 3 |
| TU | 13 | 33 | 0 | 0 | 9 | 20 | 1 | 6 | 14 | 44 | 6 | 20 |
| MS | 0 | 0 | 0 | 0 | 0 | 0 | 17 | 6 | 0 | 0 | 10 | 3 |
| SQ | 20 | 17 | 6 | 17 | 11 | 13 | 0 | 0 | 5 | 11 | 2 | 3 |
| NF | 0 | 0 | 0 | 0 | 0 | 0 | 7 | 17 | 10 | 33 | 7 | 20 |
| TF | 0 | 0 | 0 | 0 | 0 | 0 | 0 | 0 | 2 | 11 | 1 | 3 |
| UN | 0 | 0 | 17 | 33 | 9 | 13 | 8 | 6 | 41 | 33 | 20 | 17 |

*BH = ballyhoo, FF = flying fish, DF = dolphinfish, TU = Tuna, MS = mackerel scad, SQ = squid, NF = needlefish, TF = Triggerfish, UN = unidentified. Birds of unknown sex = 3x red-footed booby, 3x brown booby.
